# An Updated Definition of “Healthy” Foods in the United States: How Do They Measure in Nutrient Density, Cost, and Frequency of Consumption?

**DOI:** 10.1016/j.cdnut.2025.107545

**Published:** 2025-09-03

**Authors:** Kayla Hooker, Namrata Sanjeevi, Pablo Monsivais

**Affiliations:** Department of Nutrition and Exercise Physiology, Elson S. Floyd College of Medicine, Washington State University, Spokane, WA, United States

**Keywords:** food policy, food labels, dietary guidelines, nutrient density, cost, frequency of consumption

## Abstract

**Background:**

In 2024, the Food and Drug Administration (FDA) finalized an update to the definition of the term “healthy” as used on food labels, to align with current dietary guidelines. A holistic understanding of food choice is vital for socially- and economically-conscious food programs and policies.

**Objectives:**

This study applied the new “healthy” criteria to a nationally-representative database of foods and beverages and compared qualifying items to those that failed to qualify, in terms of nutrient density, monetary cost, and frequency of consumption.

**Methods:**

Nutrient profile scores based on the Nutrient Rich Foods Index 9.3 model and weighted frequency of consumption data from the Food and Nutrient Database for Dietary Studies were linked to data for monetary cost, based on national food prices.

**Results:**

The analysis included 3062 foods and beverages in 12 food groups, of which 14% qualified as “healthy.” Many foods did not qualify due to excess sodium and saturated fat. Overall, qualifying foods and beverages had a higher median nutrient density and frequency of consumption (*P* < 0.001) and lower median cost per serving (*P* < 0.001) compared with items that failed to qualify. Among food groups, qualifying plant protein foods, consisting primarily of nuts and seeds, were significantly lower in nutrient density and more expensive than not qualifying plant proteins. Qualifying mixed dishes were also significantly more costly than those that failed to qualify.

**Conclusions:**

Foods and beverages that would qualify as “healthy” under new FDA criteria were more nutrient dense. Overall, qualifying items may be less expensive and consumed more frequently than not qualifying items, although results differed for individual food groups. To increase the availability of foods and beverages qualifying as “healthy,” industry reformulation of packaged and processed nutrient-dense foods should be considered to reduce the addition of sodium, sugar, and saturated fat.

## Introduction

Food package labels are one approach intended to aid consumers in dietary decision-making. The requirements for the back-of-pack nutrition facts panel were last updated in 2016 to reflect updated information about certain nutrients, such as fiber and sodium, and changes in serving sizes, as well as a change in design to make information more accessible; for example, by increasing font size for the number of calories and adding a footnote to explain percent daily value [1]. Those changes were required to be reflected on food packages starting in 2020 [1]. Information on the labels, such as the number of calories or nutrients per serving, is intended to enable consumers to make more informed and, ideally, healthier food choices. Food labels may also contain health claims, such as “adequate calcium may reduce the risk of osteoporosis,” and nutrient content claims, such as “low sodium,” provided that the foods meet the Food and Drug Administration (FDA) standards for making such claims [2]. Including these claims on package labels aims to make nutrition information more accessible to a wider population; the simple and direct messaging can help consumers to make appropriate food choices quickly and without needing to understand the more in-depth information on the nutrition facts panel [3,4].

Labels may also claim that the food is “healthy,” indicating that the food contains healthy amounts of certain nutritional components [5]. The definition of “healthy” for these nutrient-based claims was first established in 1994 according to the Nutrition Labeling and Education Act of 1990 [6]. In 2022, however, the FDA proposed a new rule to update the definition of “healthy” to better reflect current dietary guidance [6]. The rule was made final on 27 December, 2024 and was initially intended to go into effect on 25 February, 2025 [6]. That effective date, however, was then postponed until 28 April, 2025 [6]. Whereas the previous requirements focused on whether a product contained certain amounts of specific, individual nutrients, the update emphasizes whether foods will contribute to an overall healthy diet pattern, while maintaining the previous requirement that foods must not exceed specified limits for sodium, saturated fat, and added sugar. If a food or beverage product meets the criteria for the definition of “healthy,” then the term “healthy” may be included in the food label, which serves to signal to consumers that the product contributes to a nutritious diet consistent with current dietary guidelines [6].

Under the new requirements, a food must contain a specific portion per serving, determined by the Reference Amount Customarily Consumed (RACC), of ≥1 of the food groups that are recommended by the Dietary Guidelines for Americans (DGA)—fruit, vegetables, whole grains, dairy, and protein foods [6]. Notably, any whole, raw fruits and vegetables and coffee, tea, or water with fewer than 5 calories per serving may automatically bear the claim “healthy” without further restrictions [6]. Limits for sodium, saturated fat, and added sugar vary depending on the type of food in question. For example, fruit and vegetable products should contain no >5% of the daily value of saturated fat, but dairy products may contain no >10% of the daily value of saturated fat [6]. Each of these requirements is based on RACC values for determining the serving size, which is the amount of the given product that people most often tend to consume in a single sitting [7].

Updating the definition and criteria for “healthy” foods marks a key step toward helping consumers identify healthy food choices based on current nutrition science and federal guidelines. However, food choices are also influenced by socioeconomic and individual factors such as cost, access, preference, and personal values, which must be considered to make realistic and impactful food purchasing recommendations [8]. Taste and affordability are noted priorities in making food purchasing decisions in the United States [9]. Particularly among low-income consumers, who tend to spend a greater share of their income on food than high-income counterparts, an increase in price can be a major deterrent to purchasing healthy food [10]. Furthermore, prioritizing the taste and cost of foods, rather than nutritional value, has previously been linked to poorer overall diet quality [11]. The purpose of this study was to apply the FDA’s updated “healthy” criteria to a nationally-representative database of foods and beverages and determine how foods that qualify under the new “healthy” criteria compare to those that fail the criteria, in terms of nutritional quality, monetary cost, and frequency of consumption.

## Methods

### Food and nutrient data

The Food and Nutrient Database for Dietary Studies (FNDDS) corresponds to What We Eat in America (WWEIA), the dietary component of the NHANES. The FNDDS provides detailed nutrient profiles for foods and beverages from WWEIA to aid dietary surveillance and research [12]. The Food Patterns Equivalents Database (FPED) converts items from FNDDS into USDA food patterns components, measured as cup equivalents for fruit, vegetables, and dairy; ounce equivalents of grains and proteins; teaspoon equivalents of added sugars; gram equivalents of solid fats and oils, and the total number of alcoholic drinks [13]. The 2017–2018 cycle of FNDDS and FPED was used to be consistent with the WWEIA cycle used for cost data estimates. Food prices were accessed via the USDA’s Purchase to Plate Suite (PP-Suite) [14], which derives its list of products from the 2017 to 2018 WWEIA survey.

**FIGURE 1:**
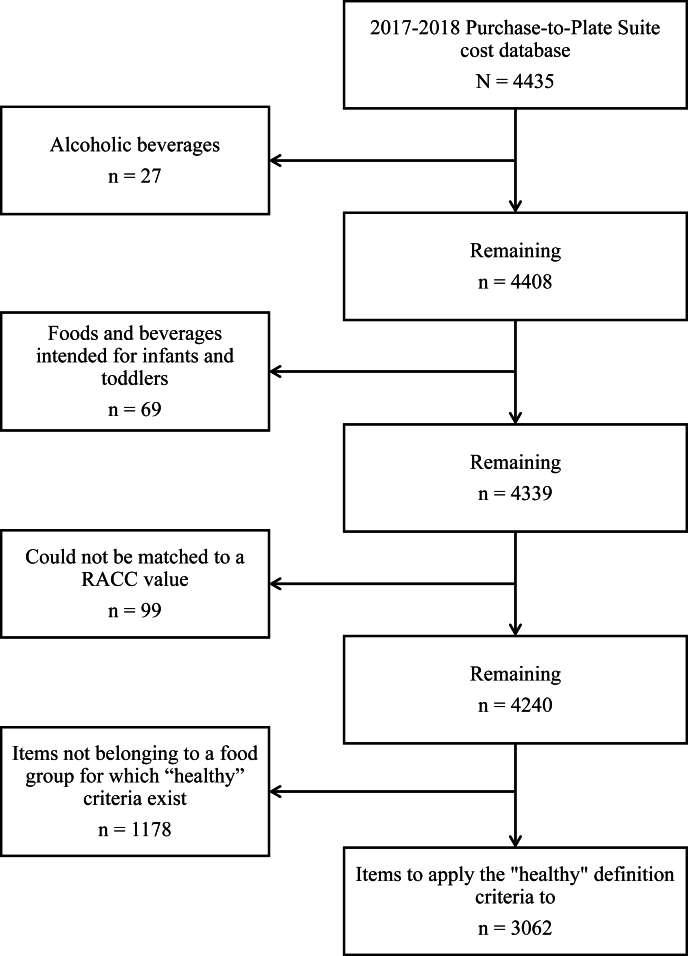
Flow chart of foods and beverages excluded from the main analysis. RACC, Reference Amount Customarily Consumed.

This study analyzed 4435 foods and beverages contained in PP-Suite dataset for the years 2017–2018, which corresponds to the 2017–2018 WWEIA cycle. Foods and beverages included in the PP-Suite dataset meet a minimal consumption threshold of being reported ≥10 times during the survey [14]. Therefore, we started with these 4435 foods as they are nationally representative of the most frequently consumed foods in the United States. Of the 4435 products, this study excluded alcoholic beverages (*n* = 27) and foods and beverages intended for infants and toddlers (*n* = 69) from the analysis. Additionally, because the criteria for the “healthy” definition depend upon the RACC value for each item, items for which an RACC value could not be determined were also excluded (*n* = 99). This final group comprised mostly items for which the portion size in FNDDS was a nonconvertible unit, such as “1 sandwich” or “1 cone,” which could not be matched to volume-based RACC values. This left 4240 foods in the analysis, summarized in Figure 1.

### FDA criteria for identifying qualifying foods and beverages

The FDA’s new criteria include specific requirements for different food groups: raw, whole fruit or vegetables; vegetable products; fruit products; grain products; dairy products; protein foods; 100% oil; oil-based spreads; oil-based dressings; mixed products; main dishes; and meal products [6]. Any items that did not meet the definition of one of these food groups were excluded from this main analysis (*n* = 1178), also shown in Figure 1. For items that did belong in one of the food groups, to be “healthy,” a food must contain a specific portion per serving, quantified by RACC, of ≥1 of the food groups recommended by the DGA—fruits, vegetables, whole grains, dairy, and protein foods [6]. Additionally, there are limits for sodium, saturated fat, and added sugar per serving. Specific criteria are also designated for certain items to be automatically considered “healthy,” including whole, raw fruits and vegetables and beverages with fewer than 5 calories [6]. The criteria are summarized in Table 1 [6,15].

**TABLE 1:** Healthy definition criteria. Foods and beverages that do not exceed limits for added sugar, sodium, and saturated fat, and also contain at least the minimum food group equivalent qualify as “healthy”.

Food group or subgroup	Added sugar limit (%MRV^1^)	Sodium limit (%MRV^1^)	Saturated fat limit (%MRV^1^)	Minimum food group equivalent
Coffee, tea, and water	—	—	—	<5 calories per serving
Raw, whole fruit or vegetables^2^	—	—	—	No additional criteria
Vegetable products	2	10	5	1/2 cup equivalent
Fruit products	2	10	5	1/2 cup equivalent
Grain products	10	10	5	3/4 ounce equivalent
Dairy products	5	10	10	2/3 cup equivalent
Protein foods				
Game meats	2	10	10	1 1/2 ounce equivalent
Seafood	2	10	5, excluding saturated fat inherent in seafood	1 ounce equivalent
Egg	2	10	10	1 ounce equivalent
Beans, peas, and lentils	2	10	5	1 ounce equivalent
Nuts, seeds, and soy products	2	10	5, excluding saturated fat inherent in nuts, seeds, and soybeans	1 ounce equivalent
100% oil	0	0	20	—
Oil-based spreads	0	5	20	—
Oil-based dressings^3^	2	5	20	—
Mixed products	10	15	10	1 FGE with ≥1/4 FGE from ≥2 food groups
Main dish^4^	15	20	15	2 FGE with ≥1/2 FGE from ≥2 food groups
Meal product^5^	20	30	20	3 FGE with ≥1/2 FGE from ≥3 food groups

Abbreviation: FGE, food group equivalents.

1 %MRV = percent of the maximum recommended value by dietary guidelines for a given nutrient.

2 “Raw, whole fruit or vegetables” does not include fruit and vegetables that have been processed, such as canned, frozen, dried, or pureed.

3 Oil dressings are defined as being composed of ≥30% oil and saturated fat must be <20% of the total fat [6] .

4 Main dishes were additionally identified by weight of ≥6 oz per serving. Further definition exists [15] but was not applied in this analysis.

5 Meal products were additionally identified by weight of ≥10 oz per serving. Further definition exists [15] but was not applied in this analysis.

RACC values are established under the FDA’s Code of Federal Regulations (CFR), Chapter 21 [7]. The food categories in the FNDDS and RACC assignment do not align; therefore, each item from FNDDS needed to be matched to an appropriate RACC. Published RACC values did not include 500 unique meat products, so typical serving sizes consumed by adults were obtained from the first 24-h dietary recall in NHANES 2017–2018. The median serving size (in grams) for each item was calculated, excluding zero values, to estimate amounts typically consumed.

**FIGURE 2:**
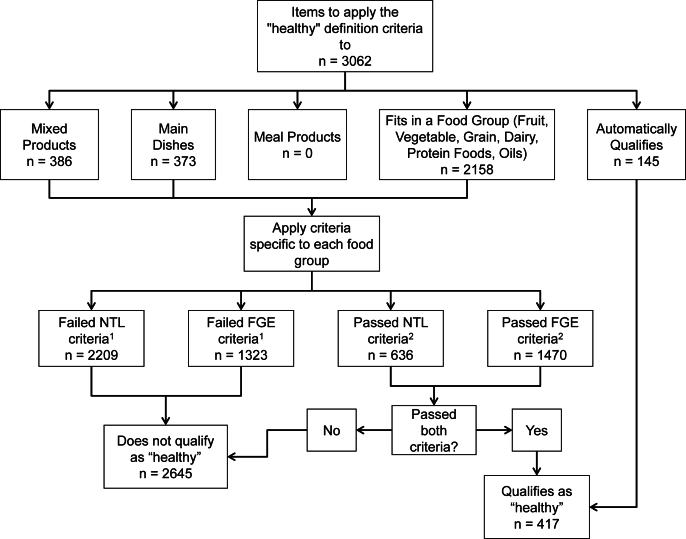
Flow chart of the process to apply the “healthy” definition criteria. NTL, nutrients to limit (added sugar, sodium, and saturated fat); FGE, food group equivalents. ^1,2^Potential for some items to appear in both groups.

The food group equivalents (FGE) data from FPED and nutrient profile data from FNDDS were used to apply the “healthy” definition criteria and determine if foods and beverages could be categorized as qualifying, or as not qualifying if they did not meet the criteria. The process is shown in Figure 2.

### Food groups

The FDA criteria aggregate many foods and beverages into broad categories. To maintain more specificity in the metrics of different foods and beverages, food groups were used based on those assigned to each item in WWEIA, resulting in 12 food groups. The 12 food groups used were: fruits, including processed fruit (frozen, canned, dried, etc.) and 100% fruit juice; vegetables, including processed vegetables (frozen, canned, dried, etc.), 100% vegetable juice, and potatoes, but excluding plant proteins; grains; dairy, including dairy alternatives; animal protein; plant protein; oils, including 100% oil, oil-based dressings, and oil-based spreads; mixed dishes; snacks; desserts; coffee, tea, water; and other beverages, which was comprised mostly of smoothies. A more detailed list of the types of items included in each food group is provided in Supplemental Table 1. The “healthy” definition does not include criteria for most beverages; therefore, the only beverages included were coffees, teas, water, and beverages that could be considered a mixed product.

#### Applying the criteria

The criteria were first applied to items that could automatically qualify as “healthy” under the new criteria (n = 145). We then assigned foods and beverages to the appropriate food group for which the “healthy” criteria exist (i.e., fruit products, vegetable products, grain products, dairy products, protein foods, oils, mixed products, main dishes, and meal products). Mixed products, main dishes, and meal products are foods that are composed of >1 ingredient and are primarily differentiated by the quantities of food they contain. Mixed products have no further definition beyond containing >1 ingredient, but have the smallest requirements for FGEs among the 3 types of combination foods. In addition to the different requirements for FGEs, main dishes must weigh ≥6 ounces per serving and contain ≥2 40-g portions of food from ≥2 different food groups of grains, fruits and vegetables, dairy, and proteins; meanwhile mixed products contain more food, needing to be ≥10 ounces per serving and containing ≥3 40-g portions from ≥2 of the same 4 food groups. The full definitions for what constitutes a main dish and meal product are defined in 21 CFR 101.13 [15]. We applied these definitions with the exception of the weight of portions from various food groups that must be included (i.e., we did not check whether foods contained the aforementioned 2 or 3 40-g portions of foods from the 4 food groups to be considered a main dish or meal product), due to practical constraints, as data were not available for the systematic and accurate conversion of FPED units to grams. Therefore, by not applying the entire definition of these food groups, we may overestimate the number of items belonging to these categories. The criteria for added sugar, saturated fat, and sodium, according to individual food groups, were applied, and the criteria for minimum FGEs, also specific to each food group, were applied separately.

#### Special cases

The final rule for the updated “healthy” definition criteria recognized that some foods have particularly small serving sizes for which the criteria would be inappropriate, for example, nuts, cookies, and pickled vegetables which all have serving sizes of 30 g, and that some foods—namely nuts, seeds, soybeans, and seafoods—inherently contain larger amounts of saturated fats but are still components of a healthy dietary pattern. For foods with small serving sizes, defined as RACC values <50 g (or 3 tablespoons), instead of applying the criteria per RACC, they are applied per 50 g.

For foods containing nuts, seeds, soybeans, and seafoods, the criteria are applied as usual, but the limit for saturated fats excludes any saturated fat inherent in the nut, seed, soybean, or seafood ingredients; therefore, it applies only to *added* saturated fat. Due to practical constraints in applying this special criterion to individual items at scale, a sensitivity analysis was performed to test what differences would be created if all the saturated fats for these foods were considered exempt from the limitation compared with having the standard criteria applied. If all saturated fats in the “Nuts, Seeds, Soy,” “Seafood,” “Seafood mixed dishes,” “Peanut butter and jelly sandwiches,” and “Egg rolls, dumplings, sushi” WWEIA categories (the last 3 of which were the only mixed dishes expected to contain an appreciable amount of nuts, seeds, soybeans, or seafood) were excluded from the limitation, an additional 106 of 1030 items would qualify as “healthy” compared with having the normal limitations applied. A brief investigation into the ingredient lists for these items revealed that vegetable oil appears to be the most common source of saturated fats outside of the nuts, seeds, soy, and seafood ingredients. The vegetable oil referenced in FNDDS contains 13.7 g of saturated fat per 100 g [12], which is relatively little compared with other oils in the database such as soybean oil (15.7% saturated fat), peanut oil (16.9% saturated fat), or coconut oil (83.25% saturated fat). Additionally, because the oils were added in small amounts relative to other ingredients containing saturated fat, and thus contributed relatively little to the total saturated fat of the item, it was assumed that all saturated fat in these 5 WWEIA categories was intrinsic to the products (not added) and therefore exempt from the limitation.

There were 1178 items that did not appropriately fit in one of the 13 food groups for which the “healthy” definition criteria exist, listed in Table 1, either because they did not satisfy the definitions of the food groups included, or because criteria were not created for the food group they belong to. This introduced an additional 3 food groups that were not part of the main analysis: powdered beverages, protein and nutritional powder, and sauces and condiments. Examples of items that belong to these food groups are provided in Supplemental Table 1. For the 1178 foods, a sensitivity analysis was performed based only on the criteria for the nutrients to limit (NTL). Of all the criteria food groups, the strictest [2% maximum recommended value (MRV) added sugar, 10% MRV sodium, 5% MRV saturated fat] and most lenient (20% MRV added sugar, 30% MRV sodium, and 20% MRV saturated fat) criteria for the 3 NTL, sodium, added sugar, and saturated fat, were applied to the 1178 items. The number of items that passed or failed the criteria were compared, along with corresponding nutrient density, cost, and weighted frequency of consumption.

### Nutrient density, monetary cost, and frequency of consumption

#### Nutrient density

The Nutrient Rich Foods Index 9.3 (NRF 9.3) is a validated, objective measurement of nutrient density based on 9 nutrients to encourage in the diet—protein, fiber, vitamin A, vitamin C, vitamin E, calcium, iron, potassium, and magnesium—and 3 NTL—sodium, saturated fat, and added sugar [16,17]. The NRF 9.3 is calculated on a 100-kcal basis as reported previously [17]. A higher NRF 9.3 score indicates higher nutrient density, with negative scores when an item contains relatively larger amounts of NTL.

#### Monetary cost

Prices in USD were retrieved from the USDA PP-Suite [14], and include the estimated price per 100 g for 4435 items from the 2017 to 2018 FNDDS [14]. Only FNDDS items that had a direct match in this database were used in this analysis. Cost per 100 g was converted to cost per RACC for the main analysis. As comparisons of food costs are strongly affected by the choice of functional unit in the denominator [18,19], we carried out additional analysis based on different cost metrics: per 100 g, per RACC, and per 100 kcal.

#### Weighted frequency of consumption

NHANES is administered to a representative sample of the United States population; thus, frequency of consumption data from this survey may provide a reasonable estimate of the food consumption practices among United States consumers. WWEIA consists of 2 24-h recall interviews and includes a count of the total number of times each item was reported on each interview day [20]. This value is a count of occurrences and is not associated with a serving size or amount of food. The frequency of consumption for each item was calculated per person-years and survey-weighted.

### Analysis

Shapiro–Wilk tests performed for each metric across food groups and qualifying/not qualifying categorization suggested significant non-normality among all indicators. Thus, Mann–Whitney U tests were conducted to assess differences in indicator scores between qualifying and not qualifying categories overall and within each food group. A secondary analysis comparing the cost calculated by different functional units was also performed, following the same analysis plan.

Statistics were performed using RStudio software, version 2023.06.0+421 “Mountain Hydrangea” release (R base statistics functions for Shapiro–Wilk and Mann–Whitney tests). Data visualizations were created using RStudio software (“ggplot2” package).

## Results

A total of 3062 items in the FNDDS 2017–2018 were included in the main analysis. When the updated “healthy” definition criteria were applied, 417 (14%) of foods and beverages qualified as “healthy,” with the balance of items we will refer to as “not qualifying.” Of the 4240 foods and beverages analyzed, the 3062 items accounted for 69% of dietary energy and 71% of dietary mass, and the 1178 uncategorized items accounted for 31% of dietary energy and 29% of dietary mass, depicted in Supplemental Figure 1. Of the 3062 categorized items, qualifying items accounted for only 6% of dietary calories and 15% of dietary mass, and not qualifying items accounted for the majority of both the calories and mass of those foods and beverages that were consumed during the survey (Supplemental Figure 1). With the exception of fruits, within every food group, qualifying items were in the minority. Furthermore, 44% of qualifying items were in the vegetables food group; 17% were fruits; 12% were plant proteins, 9% were coffee, tea, water, and 7% were animal proteins. Grains, dairy, and mixed dishes collectively accounted for the final 10% of all qualifying items (Table 2). There were no items in the oils, snacks, desserts, and beverages groups that qualified.

**TABLE 2:** Distribution of items across food groups and qualifying/not qualifying categorization.

Food group	Overall	Qualifying	Not qualifying
	*n* (% of total)	*n* (% of food group)	*n* (% of food group)
Total	3062	417	2645
	100.00%	13.62%	86.38%
Fruits	113	72	41
	3.69%	63.72%	36.28%
Vegetables	488	182	306
	15.94%	37.30%	62.70%
Grains	425	17	408
	13.88%	4.00%	96.00%
Dairy	290	21	269
	9.47%	7.24%	92.76%
Animal protein	682	31	651
	22.27%	4.55%	95.45%
Plant protein	145	52	93
	4.74%	35.86%	64.14%
Oils	52	—	52
	1.70%	—	100.00%
Mixed dishes	704	6	698
	22.99%	0.85%	99.15%
Snacks	16	—	16
	0.52%	—	100.00%
Desserts	21	—	21
	0.69%	—	100.00%
Beverages	18	—	18
	0.59%	—	100.00%
Coffee, tea, water	108	36	72
	3.53%	33.33%	66.67%

Qualifying foods and beverages are those that meet the new criteria for the “healthy” definition, whereas not qualifying items are those that fail 1 or more of those requirements.

### Qualifying and not qualifying categories

**FIGURE 3:**
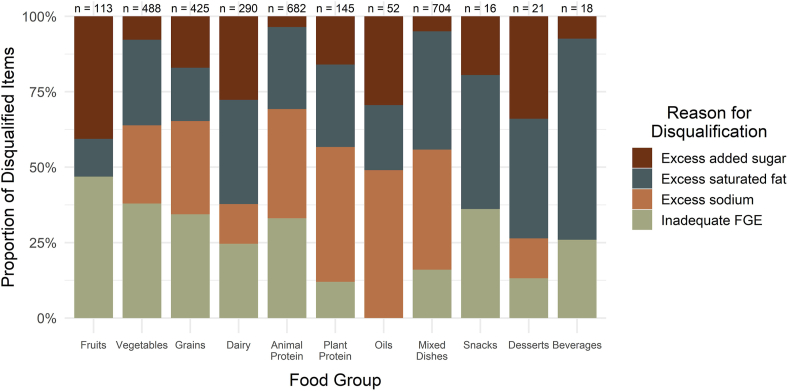
Proportion of items failing “healthy” definition criteria components by food group. Bars represent, for items that did not qualify as “healthy,” the proportion of items that were disqualified for each of 4 components of the criteria. Foods and beverages could be disqualified for any combination of the possible components; therefore, some items may be counted more than once for each food group if they were disqualified for >1 reason. N represents the total number of items in the respective food group, not the total count of each bar. FGE, food group equivalents.

Of the 3062 items in this analysis, 2645 (86%) failed to meet the updated criteria to qualify as “healthy.” Disqualification from the qualifying category could be due to failing to meet any of the 4 criteria components: *1*) inadequate equivalents of the appropriate food group per RACC; 2) excess added sugars; *3*) excess saturated fats; or *4*) excess sodium. Items could be disqualified for >1 reason, and the primary reason varied by food group, shown in Figure 3.

Among fruits, vegetables, and grains, more items failed the criteria for FGE than for the other 3 components, accounting for 27%, 38%, and 76% of each food group, respectively. Animal proteins (57%), plant proteins (46%), oils (96%), and mixed dishes (90%) failed primarily due to excess sodium, and dairy (62%) primarily failed the saturated fat criteria. All the items in the snacks, desserts, and beverages food groups also failed the saturated fat criteria.

### Nutrient density, cost, and acceptability of qualifying and not qualifying foods

Table 3 displays the medians and IQRs for nutrient density, monetary cost, and frequency of consumption, overall and by food group. The median nutrient density score, based on the NRF 9.3, among all items was 0.23. The median cost per serving was $0.52, and the median weighted frequency of consumption was 0.27 occurrences per person-year.

**TABLE 3:** Nutrient density (NRF 9.3 score), cost [USD ($) per serving, quantified by RACC], and weighted frequency of consumption (per person-year) across food groups and qualifying/not qualifying categorization.

Food group	Nutrient density (NRF 9.3)			Cost (USD) per RACC			Weighted frequency per person-year		
	Overall	Qualifying	Not qualifying	Overall	Qualifying	Not qualifying	Overall	Qualifying	Not qualifying
	Median (IQR)			Median (IQR)			Median (IQR)		
Total	0.23	0.61^∗∗∗^	0.20	0.52	0.40^∗∗∗^	0.56	0.27	0.56^∗∗∗^	0.25
	(0.12–0.43)	(0.32–1.72)	(0.1–0.36)	(0.29–1.08)	(0.25–0.58)	(0.3–1.2)	(0.07–0.81)	(0.13–1.62)	(0.06–0.74)
Fruits	0.56	0.92^∗∗∗^	0.25	0.39	0.43	0.27	0.74	1.30^∗∗∗^	0.26
	(0.25–1.1)	(0.49–1.44)	(0.12–0.45)	(0.15–0.62)	(0.15–0.64)	(0.15–0.55)	(0.24–2.63)	(0.51–4.7)	(0.08–0.62)
Vegetables	0.61	1.60^∗∗∗^	0.38	0.35	0.38	0.34	0.33	0.36	0.32
	(0.23–1.68)	(0.45–2.77)	(0.14–1.09)	(0.27–0.52)	(0.28–0.51)	(0.27–0.53)	(0.12–0.92)	(0.1–1.28)	(0.12–0.8)
Grains	0.21	0.29	0.20	0.32	0.36	0.32	0.29	1.32	0.27
	(0.12–0.4)	(0.24–0.31)	(0.12–0.45)	(0.14–0.5)	(0.25–0.5)	(0.13–0.51)	(0.07–0.95)	(0.25–2.42)	(0.07–0.87)
Dairy	0.23	0.62^∗∗∗^	0.19	0.51	0.39	0.51	0.48	0.23	0.50
	(0.04–0.43)	(0.54–0.75)	(0.03–0.37)	(0.34–0.75)	(0.32–0.75)	(0.34–0.75)	(0.11–1.5)	(0.12–0.68)	(0.1–1.54)
Animal protein	0.21	0.30^∗∗∗^	0.21	0.50	0.58	0.49	0.20	0.13	0.20
	(0.11–0.31)	(0.24–0.4)	(0.1–0.3)	(0.26–1.11)	(0.28–0.91)	(0.26–1.11)	(0.06–0.61)	(0.08–0.79)	(0.06–0.6)
Plant protein	0.34	0.26^∗^	0.37	0.41	0.52^∗∗^	0.37	0.41	0.55^∗^	0.25
	(0.23–0.43)	(0.22–0.42)	(0.24–0.43)	(0.3–0.56)	(0.34–0.6)	(0.25–0.5)	(0.11–0.85)	(0.21–1.44)	(0.1–0.73)
Oils	–0.12	–	−0.12	0.16	–	0.16	1.38	–	1.38
	(−0.22–0.06)	–	(−0.22–0.06)	(0.13–0.19)	–	(0.13–0.19)	(0.39–2.64)	–	(0.39–2.64)
Mixed dishes	0.18	0.66^∗∗∗^	0.18	1.40	3.33^∗∗^	1.40	0.16	0.29	0.16
	(0.1–0.28)	(0.51–0.77)	(0.1–0.27)	(0.93–2.13)	(1.97–4.62)	(0.91–2.11)	(0.04–0.48)	(0.22–0.33)	(0.04–0.48)
Snacks	0.23	–	0.23	0.43	–	0.43	0.93	–	0.00
	(0.13–0.41)	–	(0.13–0.41)	(0.34–0.76)	–	(0.34–0.76)	(0.45–1.78)	–	(0.45–1.78)
Desserts	0.03	–	0.03	1.25	–	1.25	0.11	–	0.11
	(0.01–0.07)	–	(0.01–0.07)	(0.86–1.39)	–	(0.86–1.39)	(0.05–0.21)	–	(0.05–0.21)
Beverages	0.68	–	0.68	0.98	–	0.98	0.60	–	0.60
	(0.51–0.86)	–	(0.51–0.86)	(0.79–1.08)	–	(0.79–1.08)	(0.27–1.14)	–	(0.27–1.14)
Coffee, tea, water	0.44	1.47^∗∗∗^	0.34	0.51	0.22^∗∗∗^	1.74	0.62	1.45^∗∗^	0.48
	(0.08–0.95)	(0.92–1.88)	(0.03–0.61)	(0.24–2.15)	(0.18–0.44)	(0.41–2.22)	(0.19–1.93)	(0.36–6.9)	(0.14–1.09)

Data represent median values and IQR. Qualifying foods and beverages are those that met the new requirements for the “healthy” definition, whereas not qualifying foods and beverages are those that failed to meet those requirements. Statistically significant differences in indicator scores between qualifying and not qualifying items assessed via Mann–Whitney U tests are indicated as: ^∗∗∗^*P* value < 0.001.

Statistically significant differences in indicator scores between healthy and not healthy items assessed via Mann–Whitney U tests are indicated as: ^∗∗∗^*P* value < 0.001; ^∗∗^*P* value < 0.01; ^∗^*P* value < 0.05.

Abbreviations: NRF, Nutrient Rich Foods Index; RACC, Reference Amount Customarily Consumed.

There were significant differences in the nutrient density, monetary cost, and frequency of consumption metrics between qualifying and not qualifying foods and beverages (*P* < 0.001 for all), with qualifying items having a higher median nutrient density and frequency, and lower cost per serving than not qualifying ones, also visualized in Supplemental Figure 2. Mean values for nutrition, cost, and frequency of consumption followed the same trend of qualifying items being more nutrient-dense, cheaper, and more frequently consumed, shown in Supplemental Figure 3.

Also shown in Table 3, Beverages had the highest median nutrient density score, mixed dishes had the highest median cost, and oils had the highest median frequency of consumption. Oils also had the lowest scores for both nutrient density and cost. Desserts were consumed at the lowest median frequency of consumption.

Among nearly all food groups, qualifying items had a higher median nutrient density score than not qualifying items (*P* < 0.001 for fruits, vegetables, dairy, animal protein, mixed dishes, and coffee, tea, water; *P* < 0.05 for plant proteins). Although overall, qualifying items were significantly less expensive, this was also true only for 1 food group, coffee, tea, water (*P* < 0.001). Qualifying items among mixed dishes and plant protein were significantly more expensive than not qualifying ones (*P* < 0.01 for both). Qualifying fruit, plant protein, and coffee, tea, water were consumed significantly more frequently than not qualifying items in the same food group (*P* < 0.001, *P* < 0.05, *P* < 0.01, respectively). Qualifying vegetables, grains, and mixed dishes were also consumed more frequently than their not qualifying counterparts, whereas not qualifying dairy and animal protein were consumed with greater frequencies.

To aid in illustrating relationships between the metrics, Supplemental Figures 4 and 5 show values for the NRF9.3 score for individual food and beverage items by food group plotted against cost (in USD) per serving and against weighted frequency of consumption, respectively. In each case, there was wide variation, substantial overlapping ranges between qualifying and not qualifying items, and generally no clear, strong linear correlations between nutrient density and cost or frequency of consumption for any food group.

### Secondary and sensitivity analyses

#### Cost of qualifying and not qualifying foods using different cost metrics

In addition to determining monetary cost per RACC, we compared costs on a per 100 g and per 100 kcal bases. Supplemental Table 2 displays the medians and IQRs for cost calculated per 100 kcal, per 100 g, and per serving, as quantified by RACC, overall and by food group. The median cost per 100 kcal among all items was $0.41. The median cost per 100 g was $0.64, and the median cost per serving was $0.52.

There were significant differences in the costs calculated by different functional units between qualifying and not qualifying foods and beverages (*P* < 0.001 for all), with qualifying items having a higher cost per 100 kcal and lower cost per 100 g and per serving compared with not qualifying ones, also shown in Supplemental Figure 6. Mean values for the 3 cost metrics followed the same trend of qualifying items being more expensive than not qualifying items per 100 kcal but cheaper per 100 g and per serving, shown in Supplemental Figure 7.

Also shown in Supplemental Table 2, oils had the lowest cost per 100 kcal and per serving, whereas mixed dishes had the highest cost per serving. Snacks had the highest cost per 100 g. Sauces and condiments had the lowest cost per 100 g but also the highest cost per 100 kcal.

Among every food group, qualifying items were more expensive per 100 kcal than not qualifying items (*P* < 0.001 for vegetables, mixed dishes, and coffee, tea, water; *P* < 0.05 for grains). Qualifying items in only 2 food groups, plant protein and mixed dishes, were more expensive per 100 g (*P* < 0.001 and *P* < 0.05, respectively), whereas most food groups had qualifying items that were more expensive per serving, as mentioned in Table 3 previously. Per 100 g, not qualifying dairy and coffee, tea, water were significantly more expensive than their qualifying counterparts (*P* < 0.001).

#### Sensitivity analysis using NTL criteria

For the 1178 foods and beverages not categorized in the main analysis, the strictest (2% MRV added sugar, 10% MRV sodium, 5% MRV saturated fat) and most lenient (20% MRV added sugar, 30% MRV sodium, 20% MRV saturated fat) criteria for the NTL, sodium, added sugar, and saturated fat, were applied to determine the effects that excluding these items might have had on our comparison of the nutrient density, cost, and frequency of consumption indicators. A detailed description of the results for the application of the strict and lenient NTL criteria is included in the supplemental documentation (Supplemental Tables 3–8; Supplemental Figures 8–16).

Eight food groups were determined from the 1178 items. Mixed dishes accounted for 432 items (37%), 321 were desserts (27%), 176 (15%) were snacks, 132 (11%) were sauces and condiments, 104 (9%) were beverages, 5 (<1%) were protein and nutritional powder, 5 (<1%) were powdered beverages, and 3 (<1%) were oils. When the strict NTL criteria were applied, 189 (16%) of foods and beverages passed, and with the lenient NTL criteria applied, 652 (55%) of foods and beverages passed. Supplemental Table 3 shows the distribution of foods and beverages by food group and whether they passed the strict criteria; Supplemental Table 4 shows the distribution for the lenient criteria.

For this analysis, failure to meet the criteria could be due to surpassing the limits set for added sugars, saturated fats, or sodium. When the strict NTL criteria were applied, the largest proportion of items exceeded the saturated fat limit (60% of items), whereas with the lenient NTL criteria, the largest proportion, 35% of items, exceeded the limit for added sugar. Altogether, 84% of items failed 1 or more of the strict NTL criteria and 45% failed 1 or more of the lenient criteria (Supplemental Figure 8).

#### Nutrient density, cost, and frequency of consumption of uncategorized foods and beverages

Across all 1178 items for both the strict and lenient criteria application, values for nutrient density and cost per serving were lower, whereas frequency of consumption was higher compared with values for items included in the main analysis (Table 3, Supplemental Table 5, Supplemental Table 6). When comparing the qualifying foods and beverages to those that passed the strict and lenient criteria, and not qualifying items to those that failed the strict and lenient criteria, the same trends remained. Items that passed in this analysis had lower nutrient density and cost than items that qualified under the “healthy” definition criteria, except in the case of frequency of consumption, where qualifying items from the main analysis were consumed with greater frequency than the items that passed either the strict or lenient NTL criteria. This indicates that the greater frequency of consumption noted among the total 1178 items in the secondary analysis can largely be attributed to the items that failed to meet the strict or lenient criteria.

#### Cost by different functional units of uncategorized foods and beverages

The 1178 foods and beverages were also cheaper when cost was calculated per 100 kcal, and most expensive when calculated per serving (Supplemental Tables 7 and 8). Compared with the 3062 foods and beverages in the main analysis, these items overall were cheaper per 100 kcal and per serving, but more expensive per 100 g. Furthermore, in analyses of both the strict and lenient criteria, items that passed were more expensive per 100 kcal compared with those that failed, but cheaper per serving and per 100 g compared with failing foods and beverages.

## Discussion

This study aimed to perform a preliminary application of the FDA’s updated “healthy” definition and determine how scores for nutrition, monetary cost, and frequency of consumption vary between foods that qualified as “healthy” and those that did not. Of 3062 foods and beverages included from the FNDDS, only 14% met all criteria to be classified as qualifying.

This analysis demonstrated that foods and beverages defined as “healthy” indeed have a higher nutritional quality, measured by the NRF 9.3 Index. Overall, and within most every food group, qualifying foods and beverages had significantly, and often substantially, higher NRF 9.3 scores than not qualifying foods and beverages. There were only 2 exceptions to this trend. Although grains did show a greater nutrient density for qualifying items, the difference was not significant. By contrast, plant proteins were the only group that showed a significantly poorer nutrient density for qualifying items. The plant proteins food group consisted of beans, peas, legumes, nuts, seeds, and soy products. Qualifying plant proteins consisted of more nuts and seeds, whereas not qualifying foods consisted of more beans, peas, and legumes. Considering the types of foods within each category within the plant proteins group, it was unsurprising to find that whereas the qualifying foods contained more of the beneficial nutrients factored into the NRF 9.3 score calculation, they also contained more saturated fat and more calories per 100-g portion. Foods that are higher in energy density, like nuts and seeds, will tend to score lower on the NRF 9.3 nutrient profile system, which is computed on a per-100-calorie basis [17].

Surprisingly, only 64% of fruit and 37% of vegetables satisfied the “healthy” definition requirements, even though these groups are consistently associated with healthier diets and lower risk of disease outcomes [21]. Additionally, a mere 4% of grains passed the “healthy” criteria. The FGE requirement was primarily responsible for disqualifying many fruit, vegetable, and grain products. The updated “healthy” definition criteria stipulate that only whole, raw fruits and vegetables may be automatically considered qualifying. Other fruit and vegetable products must contain ≥½ cup equivalents of fruits or vegetables per serving, and grains must contain ≥¾ ounce equivalents per serving [6]. Although the amendments made in the final rule make it less restrictive on beneficial food groups than the previously proposed version [22], we find that this FGE criteria remains responsible for eliminating many foods that would typically be considered part of a healthy diet. Across all food groups in this analysis, there were 1323 instances (43.2% of all foods and 50.2% of all not qualifying foods) where inadequate FGE per serving was one of the reasons a food or beverage was disqualified. However, inadequate FGE was not the foremost reason for disqualification across all items; that distinction belongs to excess sodium at 1630 instances (53.3% of all foods and 61.9% of not qualifying foods), followed closely by excess saturated fat at 1527 instances (49.9% of all foods and 58.0% of not qualifying foods). This may be best exemplified in the results for mixed dishes; of 704 foods in this group, 632 failed the sodium criterion, and 623 failed the saturated fat criterion, leading to a dismal 0.85% of the food group qualifying as “healthy.” Therefore, it appears that methods of food preparation and processing may bear most of the responsibility in determining a food’s status as “healthy.”

The relationship between the nutritional quality of foods and their monetary cost is well established, with numerous studies demonstrating that nutrient-dense foods and diets tend to be more expensive on a per-calorie basis than less-nutritious alternatives [[23], [24], [25], [26], [27], [28], [29]]. In the current study, food costs were expressed on a per-serving (RACC) basis, and qualifying items overall were significantly less costly than not qualifying items, consistent with similar analyses of food prices per serving [19,28,29]. However, among individual food groups, only coffee, tea, water had significantly lower costs for qualifying items. Additionally, qualifying options were substantially and significantly more costly for some food groups, namely mixed dishes and plant proteins, corroborating the trends in the literature. Trends in costs also differed depending on the functional unit by which it was calculated, consistent with previous reports [18,19,30]. Although the cost analysis on a per-100 g basis was similar to the primary analysis of cost per RACC, on a per-100 kcal basis, the cost of qualifying items was consistently more expensive than not qualifying ones. The secondary analysis of uncategorized foods and beverages yielded consistent results, where items that passed the NTL criteria were more expensive per 100 kcal, but cheaper per serving and per 100 g compared with items that failed the criteria. Food costs can be a major factor in food choices, and for low-income individuals, it can be a barrier to accessing and consuming nutritious foods [23,24,31,32]. The findings in this study continue to add support for the argument that food affordability must be addressed for improving the accessibility of nutrient-dense diets, whereas also suggesting that it is possible among certain food groups that healthier food options can be less expensive.

This study also analyzed the frequency of consumption as a population-level indicator of the contribution of these foods to the American diet. The analysis revealed the mean frequency of consumption of qualifying foods and beverages was significantly greater than not qualifying items; 3 food groups (fruits, plant protein, and coffee, tea, water) additionally showed a significantly greater median frequency of consumption among qualifying items, with vegetables, grains, and mixed dishes also having a similar, but not significant, result. This contrasts with literature showing that less expensive, less nutrient-dense foods tend to be consumed more often [23,24]. Qualifying fruits and coffee, tea, water had 2 of the highest nutrient density scores and frequencies of consumption; coffee, tea, and water were also the cheapest per serving among qualifying items, whereas fruits were more moderately priced. These 2 groups may present a prime example of an opportunity for frequently consumed foods to contribute to a more nutrient-dense diet with a relatively low price tag, particularly when only considering qualifying foods and beverages in these groups. Qualifying vegetables were likewise cheap and nutrient dense, yet the frequency by which this group was consumed might suggest that the opportunity is missed by many individuals. However, if one were looking to substitute a qualifying item for a not qualifying counterpart in the same food group, it likely will be more expensive.

The FDA’s update to the “healthy” definition intends to help people meet current dietary pattern guidelines through food choices [6]. Ideally, the purchase and consumption of foods and beverages bearing the “healthy” label claim will help consumers meet dietary recommendations for a more nutrient-dense diet. However, food choices also rely upon factors other than nutrition, and based on this analysis, qualifying foods and beverages were not lower cost or more frequently consumed for every food group. If consumers base food choices on the “healthy” claim, they may be likely to achieve their nutritional requirements, but they potentially would need to deviate from their typical dietary patterns or pay more for that food.

To effectively accomplish the goals of a “healthy” food label, we must understand why many foods and beverages known to be part of a healthy diet failed to be classified as healthy. A substantial proportion of products were disqualified from being considered “healthy” due to excess sodium and saturated fat. One promising action to widen the pool of healthy foods involves industry reformulation of packaged and processed foods. Reducing the sodium, saturated fats, and sugars added during processing would allow many items, such as canned fruits, vegetables, and beans, to avoid exclusion from the qualifying classification. For instance, many canned beans were disqualified from the healthy category due to excess saturated fat and/or sodium, even though beans are naturally low in these nutrients. Previous studies have shown that product reformulation may indeed reduce intake of nutrients to limit [33,34] such as sodium, saturated fat, and added sugar. However, although reformulations have the potential to make adherence to certain dietary guidance more likely, these changes are not guaranteed to also improve overall dietary quality [35]. Product reformulation is also limited by research and development needs, available processing methodologies, and may result in a greater cost to consumers [36,37], all hurdles that would need to be overcome if reformulation is to be a major avenue of increasing the availability of “healthy” foods. Finally, government programs can be pivotal in increasing the affordability and consumption of healthy foods. One such program, the Supplemental Nutrition Assistance Program (SNAP) Healthy Incentives program, incentivizes the purchase of healthy foods, which are defined by specific eligibility criteria [38], similar to the “healthy” definition, and saw a marked increase in the consumption of fruits and vegetables during the pilot program [38]. However, for these programs to be maximally effective, the eligibility criteria must be appropriate for the referenced serving sizes, and the availability of qualifying foods must not be severely limited by food processing methods.

### Limitations and methodological considerations

Some limitations of this study are related to the lack of direct linkage between the databases used. First, price data did not contain an exact match for every item in the FNDDS database, limiting the number of foods and beverages that could be accurately analyzed. Second, the updated healthy definition necessitates the use of RACC values as a functional unit; however, these values were not available for every item included in this analysis. An estimated RACC was calculated to fill in the gaps; however, our estimation methods may have differed from those used by the FDA. Furthermore, the accessible RACC values needed to be manually matched to the FNDDS items and converted to appropriate units, with each step introducing an additional possibility of error. A more standardized bridge between these datasets would moderate many of these limitations.

It is important to recognize that the exclusion of foods and beverages from the main analysis, including energy-dense foods and sugar-sweetened beverages, may impact the overall results. A sensitivity analysis was performed on these items that were not included in the main analysis because they did not appropriately fit in one of the food groups for which the “healthy” criteria are defined. This analysis revealed that the uncategorized items had an overall lower median nutrient density, lower median cost, and higher median frequency of consumption compared with items included in the study. Additionally, among the uncategorized items, those that passed either NTL criteria tended to be more nutrient-dense and less expensive, but also consumed less frequently than items that failed the NTL criteria. Thus, exclusion of these items may have biased the representation of foods and beverages in the analysis, particularly by inflating the average nutrient density and monetary cost of foods and beverages.

Finally, as the “healthy” definition rules were not necessarily intended to be applied to foods in a database at scale, there were practical limitations in applying some of the more nuanced criteria to this dataset. In particular, the lack of detailed ingredient information (or any ingredient information at all) for many products in the database prevented us from applying the criteria for excluding saturated fats inherent in nuts, seeds, soybeans, and seafood as intended. For the same reason, we could not apply an additional criterion that stipulated individual foods or mixed products composed of only the food groups to be encouraged [5] with no additional ingredients except for water, be automatically considered “healthy” with no other limitations or criteria because of their ability to contribute to a healthy dietary pattern. Inconsistencies such as these in the database add further complexities to applying the criteria and may necessitate manually sorting through ingredients for foods and beverages, which, for this preliminary application, was not feasible. The exclusion of these two criteria from our application then means our analysis may be overestimating the number of qualifying items based on saturated fats, and underestimating the number of qualifying items based on their individual ingredients.

In conclusion, this study assessed how the FDA’s updated definition of “healthy” relates to measures of nutrient density, food cost, and frequency of consumption—each being a vital component of diets for a healthier population. Results showed that the vast majority of foods and beverages were not qualifying, highlighting the importance of increasing access to healthy foods in the food system. This analysis also demonstrated that foods and beverages defined as “healthy” indeed have a higher nutritional quality, and whereas they were also cheaper per serving and consumed more frequently overall, this was not always the case for individual food groups. If consumers base food choices on the “healthy” claim, they may be likely to achieve their nutritional requirements and may not need to deviate drastically from their typical dietary patterns within certain food groups, but they may still need to pay more for that food.

## Author contributions

The authors’ contributions were as follows – KH, PM: designed research and wrote paper; KH, NS conducted research; KH: analyzed data and had primary responsibility for final content; and all authors: read and approved the final manuscript.

## Data availability

Data sources described in the manuscript are publicly and freely available without restriction at: https://www.federalregister.gov/documents/2025/02/25/2025-03118/food-labeling-nutrient-content-claims-definition-of-term-healthy (Accessed 2025 Apr 8) (healthy definition criteria); https://www.ecfr.gov/current/title-21/chapter-I/subchapter-B/part-101/subpart-A/section-101.12 (Accessed 2025 Apr 8) (RACC values); https://www.ars.usda.gov/northeast-area/beltsville-md-bhnrc/beltsville-human-nutrition-research-center/food-surveys-research-group/docs/fndds-download-databases/ (Accessed 2025 Apr 8) (FNDDS); http://ars.usda.gov/northeast-area/beltsville-md-bhnrc/beltsville-human-nutrition-research-center/food-surveys-research-group/docs/fped-databases/ (Accessed 2025 Apr 8) (FPED); https://www.ers.usda.gov/data-products/purchase-to-plate (Accessed 2025 Apr 8) (PP-Suite). Generated data described in the manuscript, code book, and analytic code will be made available on request pending approval.

## Funding

The authors reported no funding received for this study.

## Conflict of interest

The authors report no conflicts of interest.
