# Supplementary material for: An Updated Definition of “Healthy” Foods in the United States: How Do They Measure in Nutrient Density, Cost, and Frequency of Consumption?
Source: Curr Dev Nutr. 2025 Sep 3;9(10):107545. doi: 10.1016/j.cdnut.2025.107545 (PMC12554928; doi:10.1016/j.cdnut.2025.107545)
Supplement: Multimedia component 1 [file mmc1.docx]

**An updated definition of “healthy” foods in the US: How do they measure in nutrient density, cost, and frequency of consumption?**

**Supplemental Material**

Hooker, Sanjeevi, and Monsivais

**Supplemental Table 1**. Examples of the types of foods included in each food group.

| Food Group | Types of Items^1^ |
| --- | --- |
| Fruits | 100% Fruit juice; Apples; Bananas; Blueberries and other berries; Citrus fruits; Dried fruits; Grapes; Mango and papaya; Melons; Mustard and other condiments; Other fruits and fruit salads; Peaches and nectarines; Pears; Pineapple; Strawberries |
| Vegetables | 100% Vegetable juice; Broccoli; Cabbage; Carrots; Coleslaw; Corn; French fries and other fried white potatoes; Fried vegetables; Lettuce; Mashed potatoes and white potato mixtures; Pickled vegetables; Onions; Other dark green vegetables; Other red and orange vegetables; Other starchy vegetables; Spinach; String beans; Tomatoes; Vegetables on a sandwich; White potatoes, baked or boiled |
| Grains | Bagels and English muffins; Biscuits, muffins, quick breads; Grits and other cooked cereals; Not included in a food category; Oatmeal; Pancakes, waffles, French toast; Pasta, noodles, cooked grains; Ready-to-eat cereal, higher sugar; Ready-to-eat cereal, lower sugar; Rice; Rolls and buns; Tortillas; Yeast breads |
| Dairy | Cheese; Cottage/ricotta cheese; Cream and cream substitutes; Cream cheese, sour cream, whipped cream; Flavored milk, lowfat; Flavored milk, nonfat; Flavored milk, reduced fat; Flavored milk, whole; Ice cream and frozen dairy desserts; Milk shakes and other dairy drinks; Milk substitutes; Milk, lowfat; Milk, nonfat; Milk, reduced fat; Milk, whole; Yogurt, Greek; Yogurt, regular |
| Animal Protein | Bacon; Beef, excludes ground; Chicken patties, nuggets and tenders; Chicken, whole pieces; Cold cuts and cured meats; Eggs and omelets; Fish; Frankfurters; Ground beef; Lamb, goat, game; Liver and organ meats; Pork; Sausages; Shellfish; Other poultry |
| Plant Protein | Beans, peas, legumes; Nuts and seeds; Processed soy products |
| Oils | Butter and animal fats; Margarine; Mayonnaise; Salad dressings and vegetable oils |
| Mixed Dishes | Bean, pea, legume dishes; Burgers; Burritos and tacos; Cheese sandwiches; Chicken/turkey sandwiches; Egg rolls, dumplings, sushi; Egg/breakfast sandwiches; Frankfurter sandwiches; Fried rice and lo/chow mein; Meat mixed dishes; Nachos; Other Mexican mixed dishes; Pasta mixed dishes; Peanut butter and jelly sandwiches; Pizza; Poultry mixed dishes; Rice mixed dishes; Seafood mixed dishes; Seafood sandwiches; Soups; Stir-fry and soy-based sauce mixtures; Turnovers and other grain-based items; Vegetable dishes |
| Snacks | Cereal bars; Crackers, excludes saltines; Nutrition bars; Popcorn; Potato chips; Pretzels/snack mix; Saltine crackers; Tortilla, corn, other chips |
| Desserts | Cakes and pies; Candy containing chocolate; Candy not containing chocolate; Cookies and brownies; Doughnuts, sweet rolls, pastries; Gelatins, ices, sorbets; Pudding |
| Beverages | Diet soft drinks; Diet sport and energy drinks; Fruit drinks; Nutritional beverages; Other diet drinks; Other fruit juice; Smoothies and grain drinks; Soft drinks; Sport and energy drinks |
| Coffee, Tea, Water | Bottled water; Coffee; Enhanced or fortified water; Flavored or carbonated water; Tap water; Tea |
| Sauces and Condiments | Dips, gravies, other sauces; Pasta sauces, tomato-based; Soy-based condiments; Mustard and other condiments; Tomato-based condiments; Sugars and honey; Jams, syrups, toppings |
| Powdered Beverages | Milk, dry, not reconstituted; Cocoa powder, not reconstituted; Chocolate beverage powder, dry mix, not reconstituted; Milk, malted, dry mix, not reconstituted; Coffee, instant, not reconstituted |
| Protein and Nutritional Powder | Nutritional powder mix ; Nutritional powder mix, whey based, NFS; Nutritional powder mix, protein, soy based, NFS; Nutritional powder mix, protein, light, NFS; Nutritional powder mix, protein, NFS |
| ^1^Types of items correspond to the WWEIA (What We Eat In America) survey food categories from the 2017-2018 data cycle. Individual items in the database fall under each of the listed categories. | |

**Supplemental Figure 1** shows the contribution of foods and beverages to the total calories and amount in grams that were consumed. The initial sample represents foods and beverages from the PP-Suite cost database, minus the excluded foods and beverages (alcoholic beverages, foods and beverages intended for infants and toddlers, and foods for which a RACC value could not be determined), as indicated in Figure 1 of the main text. The final sample consists of items that were in the main analysis and were designated as “Qualifying” or “Not Qualifying” for the updated “healthy” definition, and the uncategorized items are those not designated as either “Qualifying” or “Not Qualifying” and were included in the secondary analyses. Of the initial sample, uncategorized foods and beverages accounted for approximately 30% of both the calories and grams of foods and beverages, meaning that, while the majority of foods by calories and weight were considered in the main analysis, approximately 30% were not. Of the items in the final sample, Qualifying items accounted for only 6% of the calories and 15% of the foods and beverages by weight, leaving Not Qualifying items to account for the majority of both the calories and amount of food.


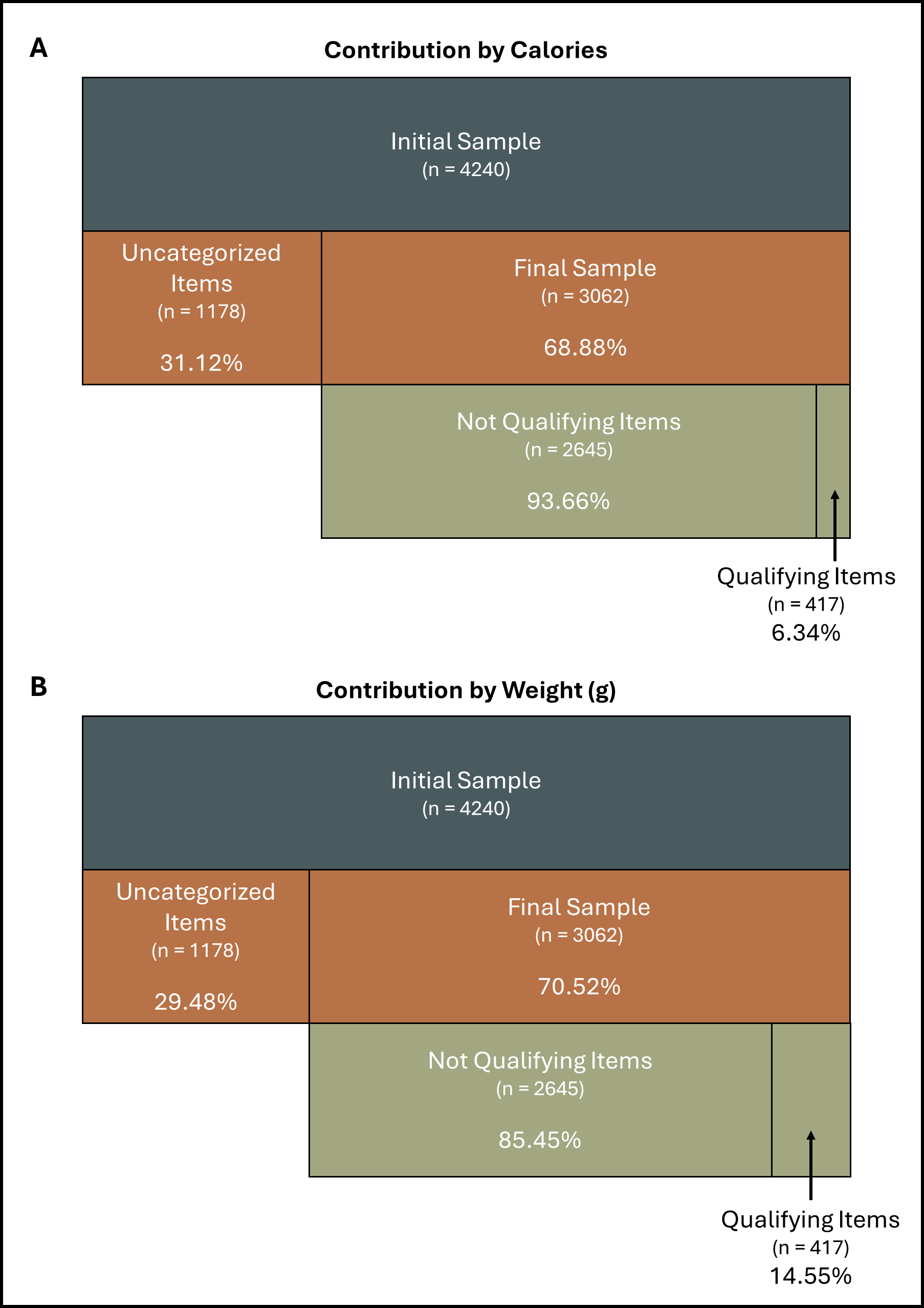


**Supplemental Figure 1**. Contributions to foods and beverages consumed by calories (a) and by weight in grams (b). Box widths correspond to proportions of the row above.


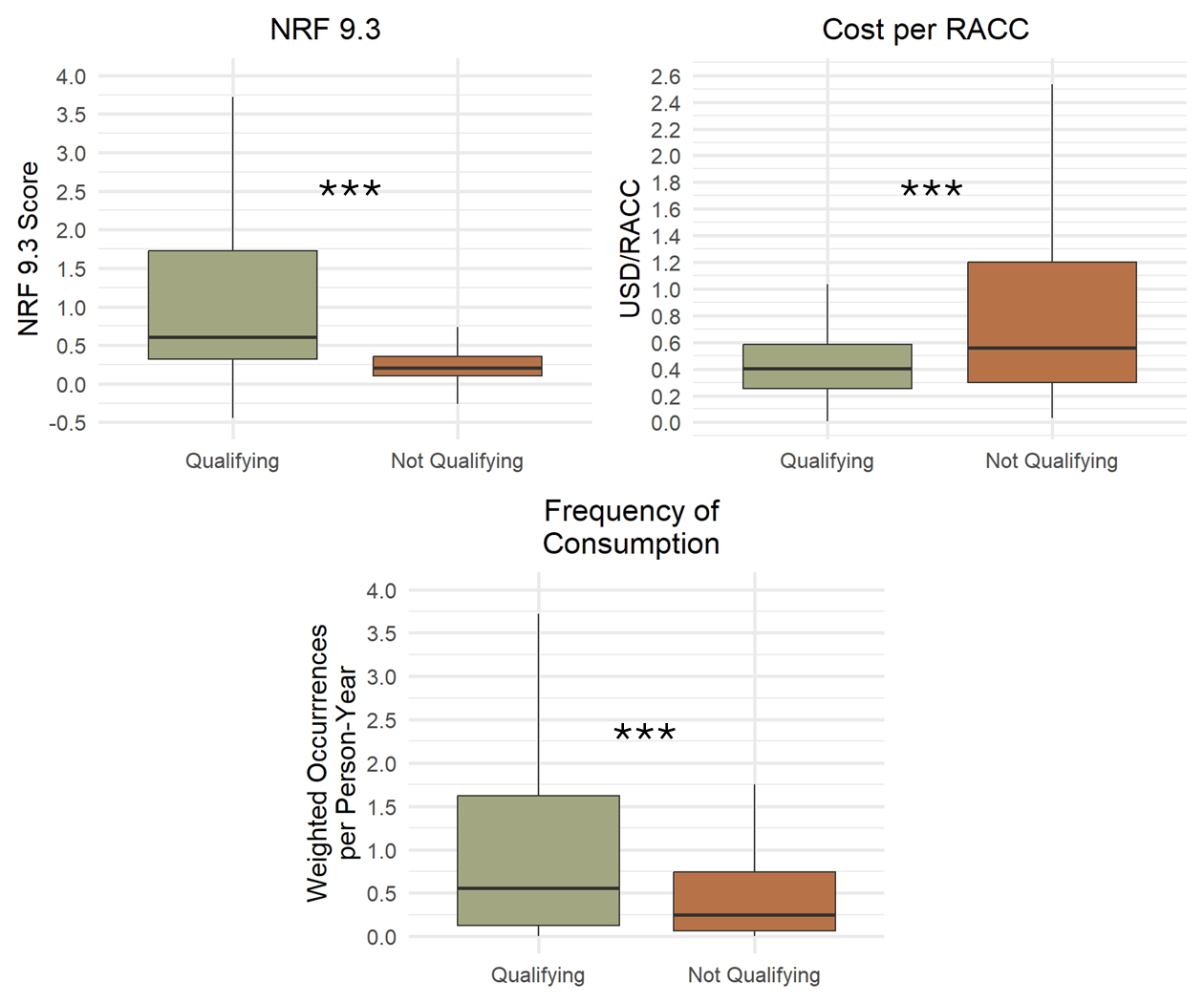


**Supplemental Figure 2**. Median indicator scores for Qualifying and Not Qualifying foods and beverages. Boxes represent median and interquartile range for nutrient density determined by NRF 9.3 scores, cost in USD per serving, and weighted frequency of consumption per person-year. Whiskers represent minimum and maximum for each indicator. Values exclude outliers, defined as exceeding 1.5 times the IQR. Foods and beverages are separated by whether they qualify under the new “healthy” definition criteria. Qualifying foods and beverages are those that meet the new criteria for the “healthy” definition, while Not Qualifying foods and beverages are those that do not. Statistically significant differences between Qualifying and Not Qualifying items assessed via Mann-Whitney U-tests are indicated as: *** p-value < 0.001. The number of outliers for each indicator are as follows: NRF 9.3: Healthy = 24 upper, Not Healthy = 9 lower/11 upper; Cost: Healthy = 32 upper, Not Healthy = 775 upper; Frequency: Healthy = 53 upper, Not Healthy = 94 upper.


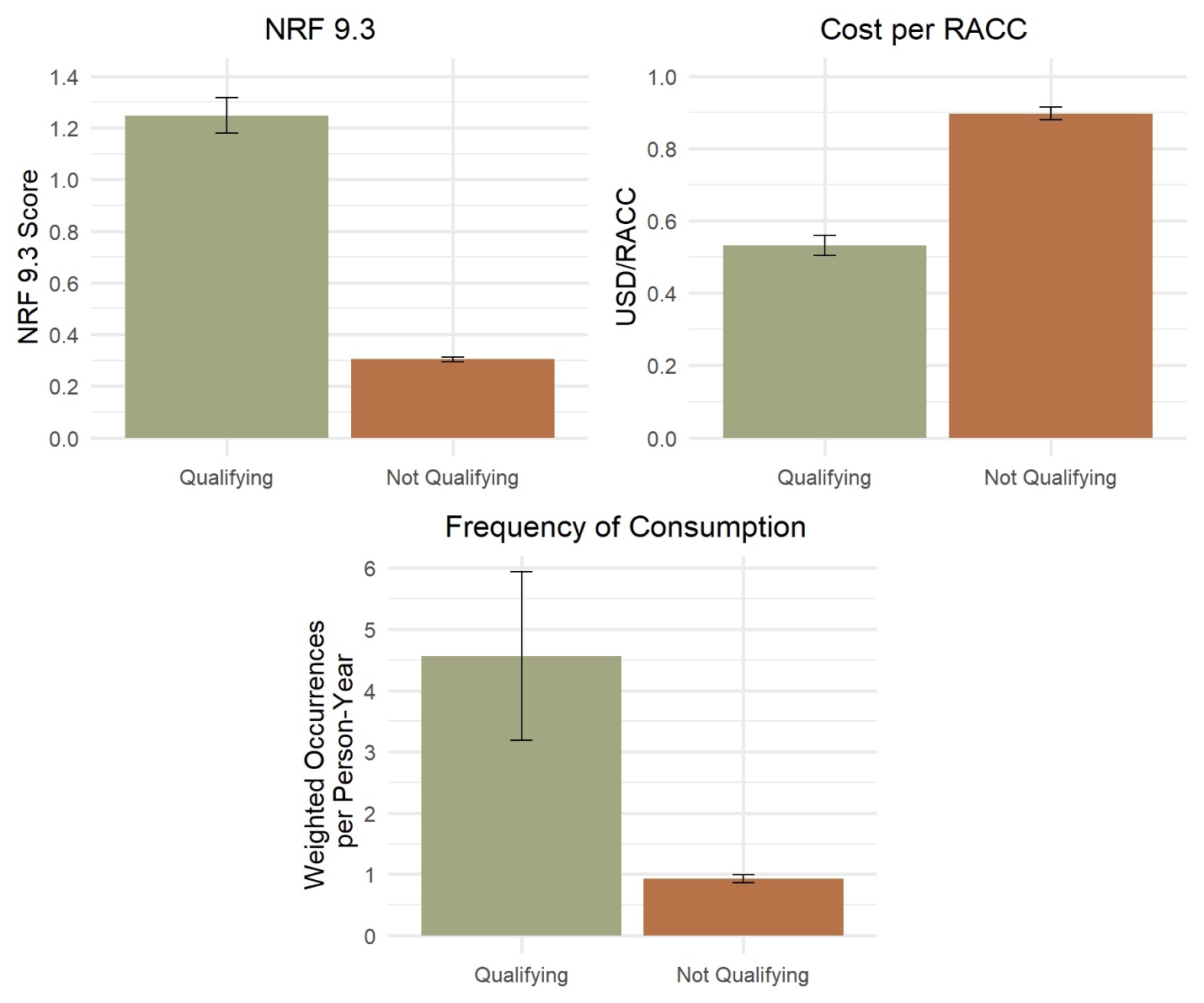


**Supplemental Figure 3.** Mean indicator scores for Qualifying and Not Qualifying foods and beverages. Data represent mean nutrient density determined by NRF 9.3 scores, cost in USD per serving, and weighted frequency of consumption per person-year. Error bars represent the standard error of the mean for each indicator. Foods and beverages are separated by whether or not they qualify under the new “healthy” definition criteria. Qualifying foods and beverages are those that meet the new criteria for the “healthy” definition, while Not Qualifying foods and beverages are those that do not.

**Supplemental Figure 4** shows values for the NRF9.3 score for individual food and beverage items by food group plotted against cost (in USD) per serving, with the color and shape of the point corresponding to whether the item qualified or did not qualify under the updated “healthy” definition criteria. Negative NRF9.3 scores are possible, indicating a lower nutrient density and greater content of nutrients to limit in the diet (saturated fat, sodium, and added sugar). In the main analysis, we found that overall, foods qualifying for the “healthy” definition had lower median costs per serving, suggesting that healthier foods may be cheaper than unhealthier options. However, significantly lower costs of Qualifying foods in the Coffee, Tea, Water food group were largely responsible for this outcome. Other food groups showed no significant different in cost or, as with Plant Proteins and Mixed Dishes, showed that Qualifying foods were more expensive per serving. The relationship between NRF9.3 scores and costs per serving for individual items, separated by Qualifying or Not Qualifying status, within each food group can be seen in **Supplemental Figure 4**, with no food groups indicating a clear linear relationship. Based on the figure, it appears that particularly among Fruits and Vegetables Qualifying items are centered more closely around higher cost and higher nutrient density values. Other contrasts in the patterns of cost and nutrient density between the Qualifying and Not Qualifying items within each food group are made more evident by the figure. For example, Qualifying items among Dairy and Mixed Dishes tend to be clustered at higher NRF9.3 scores than Not Qualifying items, while both subgroups have fairly varied costs. Among Grains and Animal Protein, both Qualifying and Not Qualifying items are centered similarly, but there is greater variability in both the nutrient density and cost of Not Qualifying items in both groups.

**Supplemental Figure 5** similarly shows values for the NRF9.3 score for individual food and beverage items by food group plotted against the weighted frequency of consumption, with the color and shape of the point corresponding to whether the item qualified or did not qualify under the updated “healthy” definition criteria. Negative NRF9.3 scores are possible, indicating a lower nutrient density and greater content of nutrients to limit in the diet (saturated fat, sodium, and added sugar). The main analysis also found that overall, Qualifying items had a greater median frequency of consumption than Not Qualifying items. This trend was also true within every food except Dairy, and was particularly strong among Fruits and Coffee, Tea, Water. In **Supplemental Figure 5** it is again apparent that there are no clear linear relationships between frequency of consumption and nutrient density among these foods. In viewing **Supplemental Figure 5**, it may appear that among Fruits, Vegetables, Plant Protein, and Coffee, Tea, Water Qualifying items may be more optimally situated at higher frequencies of consumption and higher nutrient densities than their Not Qualifying counterparts.


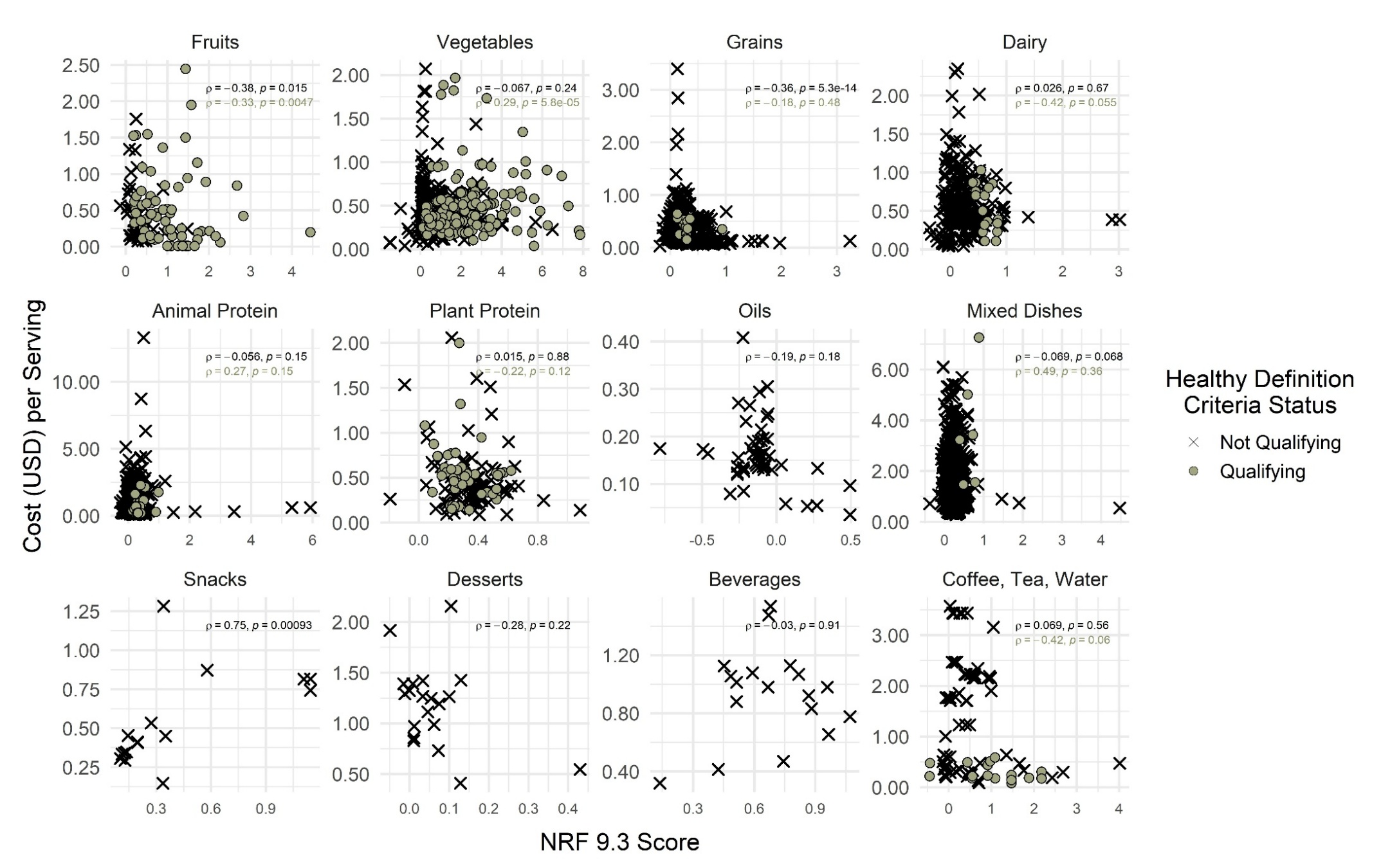


**Supplemental Figure 4**. NRF9.3 score plotted against cost per serving for individual foods and beverages by food group. Color and shape of points correspond to status of qualifying or not qualifying for the updated “healthy” definition. Spearman correlation coefficients and p-values for each group are displayed, with color representing the “healthy” definition criteria status.


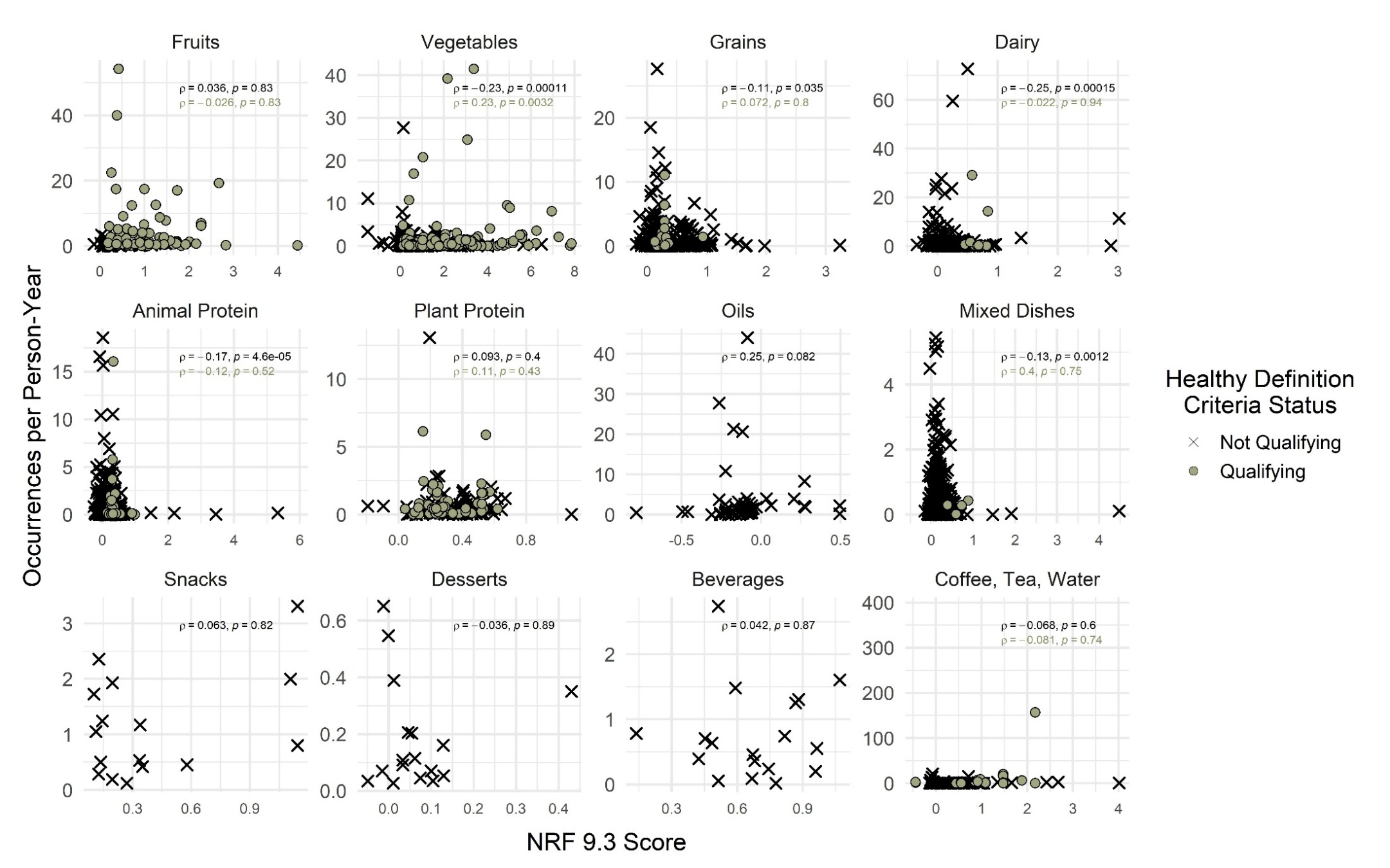


**Supplemental Figure 5**. NRF9.3 score plotted against weighted frequency of consumption for individual foods and beverages by food group. Color and shape of points correspond to status of qualifying or not qualifying for the updated “healthy” definition. Spearman correlation coefficients and p-values for each group are displayed, with color representing the “healthy” definition criteria status.

**Supplemental Table 2**. Cost (USD) calculated per 100 kilocalories, per 100 grams, and per serving, quantified by RACC, across food groups and Qualifying/Not Qualifying categorization. Data represent median values and interquartile range (IQR). Qualifying foods and beverages are those that met the new requirements for the “healthy” definition, while Not Qualifying foods and beverages are those that failed to meet those requirements. Statistically significant differences in indicator scores between Qualifying and Not Qualifying items assessed via Mann-Whitney U tests are indicated as: *** p-value < 0.001; ** p-value < 0.01; * p-value < 0.05.

| Food Group | Cost (USD) per 100kcal | | | Cost (USD) per 100g | | | Cost (USD) per RACC | | |  |
| --- | --- | --- | --- | --- | --- | --- | --- | --- | --- | --- |
|  | Overall | Qualifying | Not Qualifying | Overall | Qualifying | Not Qualifying | Overall | Qualifying | Not Qualifying | |
|  | Median [IQR] | | | Median [IQR] | | | Median [IQR] | | | |
| Total | 0.41 | 0.73*** | 0.39 | 0.64 | 0.49*** | 0.67 | 0.52 | 0.40*** | 0.56 | |
|  | [0.25-0.76] | [0.34-1.57] | [0.24-0.68] | [0.41-1.06] | [0.29-0.97] | [0.44-1.07] | [0.29-1.08] | [0.25-0.58] | [0.3-1.2] | |
| Fruits | 0.77 | 0.82 | 0.67 | 0.5 | 0.49 | 0.52 | 0.39 | 0.43 | 0.27 | |
|  | [0.45-1.27] | [0.44-1.53] | [0.46-0.94] | [0.36-0.82] | [0.28-0.77] | [0.39-0.96] | [0.15-0.62] | [0.15-0.64] | [0.15-0.55] | |
| Vegetables | 0.67 | 0.99*** | 0.52 | 0.44 | 0.44 | 0.44 | 0.35 | 0.38 | 0.34 | |
|  | [0.33-1.52] | [0.47-1.95] | [0.29-1.13] | [0.34-0.68] | [0.33-0.65] | [0.35-0.71] | [0.27-0.52] | [0.28-0.51] | [0.27-0.53] | |
| Grains | 0.2 | 0.27* | 0.20 | 0.58 | 0.49 | 0.59 | 0.32 | 0.36 | 0.38 | |
|  | [0.15-0.26] | [0.19-0.39] | [0.15-0.26] | [0.36-0.77] | [0.15-0.78] | [0.36-0.77] | [0.14-0.5] | [0.25-0.5] | [0.13-0.51] | |
| Dairy | 0.32 | 0.39 | 0.31 | 0.44 | 0.15*** | 0.46 | 0.51 | 0.39 | 0.51 | |
|  | [0.24-0.51] | [0.26-0.66] | [0.24-0.5] | [0.19-0.83] | [0.13-0.41] | [0.2-0.91] | [0.34-0.75] | [0.32-0.75] | [0.34-0.75] | |
| Animal Protein | 0.55 | 0.71 | 0.55 | 1.07 | 1.06 | 1.08 | 0.5 | 0.58 | 0.49 | |
|  | [0.34-0.96] | [0.47-1.19] | [0.34-0.95] | [0.75-1.85] | [0.54-1.69] | [0.75-1.86] | [0.26-1.11] | [0.28-0.91] | [0.26-1.11] | |
| Plant Protein | 0.26 | 0.29 | 0.22 | 0.87 | 1.72*** | 0.48 | 0.41 | 0.52** | 0.37 | |
|  | [0.17-0.35] | [0.19-0.34] | [0.15-0.37] | [0.32-1.7] | [1.07-1.99] | [0.28-1.25] | [0.3-0.56] | [0.34-0.6] | [0.25-0.5] | |
| Oils | 0.18 | - | 0.18 | 0.63 | - | 0.63 | 0.16 | - | 0.16 | |
|  | [0.12-0.37] | - | [0.12-0.37] | [0.49-0.91] | - | [0.49-0.91] | [0.13-0.19] | - | [0.13-0.19] | |
| Mixed Dishes | 0.43 | 1.11*** | 0.43 | 0.69 | 1.33* | 0.69 | 1.4 | 3.33** | 1.40 | |
|  | [0.3-0.66] | [1.06-1.53] | [0.3-0.66] | [0.51-1.03] | [0.81-1.86] | [0.51-1.03] | [0.93-2.13] | [1.97-4.62] | [0.91-2.11] | |
| Snacks | 0.28 | - | 0.28 | 1.26 | - | 1.26 | 0.43 | - | 0.43 | |
|  | [0.21-0.47] | - | [0.21-0.47] | [1.12-1.9] | - | [1.12-1.9] | [0.34-0.76] | - | [0.34-0.76] | |
| Desserts | 0.28 | - | 0.28 | 1.06 | - | 1.06 | 1.25 | - | 1.25 | |
|  | [0.24-0.32] | - | [0.24-0.32] | [0.95-1.36] | - | [0.95-1.36] | [0.86-1.39] | - | [0.86-1.39] | |
| Beverages | 0.64 | - | 0.64 | 0.43 | - | 0.43 | 0.98 | - | 0.98 | |
|  | [0.5-0.8] | - | [0.5-0.8] | [0.36-0.46] | - | [0.36-0.46] | [0.79-1.08] | - | [0.79-1.08] | |
| Coffee, Tea, Water | 1.63 | 5.10*** | 1.37 | 0.14 | 0.06*** | 0.47 | 0.51 | 0.22*** | 1.74 | |
|  | [0.83-4.32] | [4.84-8.43] | [0.66-2.19] | [0.07-0.59] | [0.05-0.12] | [0.11-0.59] | [0.24-2.15] | [0.18-0.44] | [0.41-2.22] | |

**
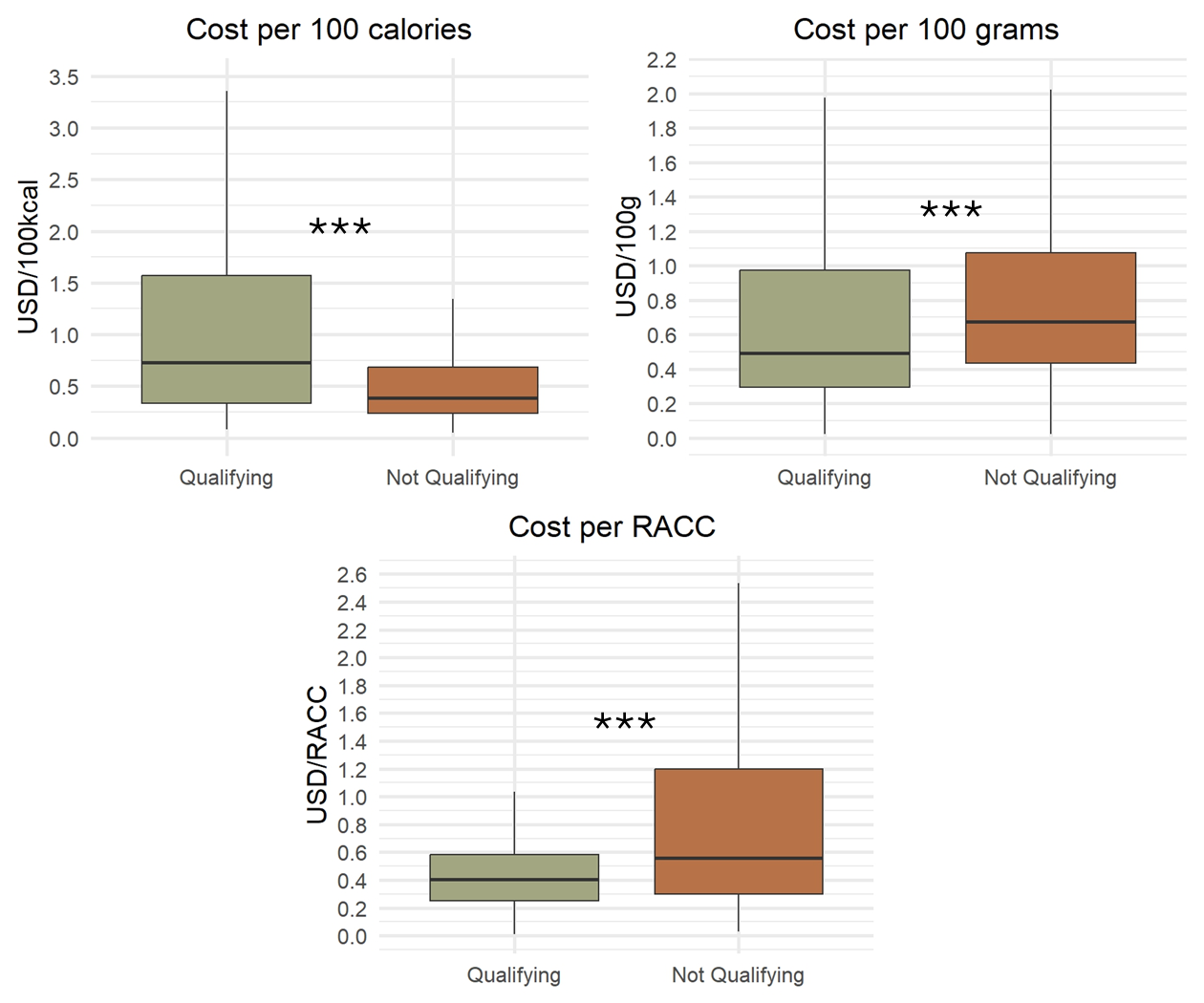
**

**Supplemental Figure 6:** Median indicator scores for Qualifying and Not Qualifying foods and beverages. Boxes represent median and interquartile range for nutrient density determined by NRF 9.3 scores, cost in USD per serving, and weighted frequency of consumption per person-year. Whiskers represent minimum and maximum for each indicator. Values exclude outliers, defined as exceeding 1.5 times the IQR. Foods and beverages are separated by whether they qualify under the new “healthy” definition criteria. Qualifying foods and beverages are those that meet the new criteria for the “healthy” definition, while Not Qualifying foods and beverages are those that do not. Statistically significant differences between Qualifying and Not Qualifying items assessed via Mann-Whitney U-tests are indicated as: *** p-value < 0.001. The number of outliers for each indicator are as follows: per 100 kcal: Healthy = 38 upper, Not Healthy = 26 lower/32 upper; per 100 g: Healthy = 30 upper, Not Healthy = 206 upper; per RACC: Healthy = 32 upper, Not Healthy = 775 upper.


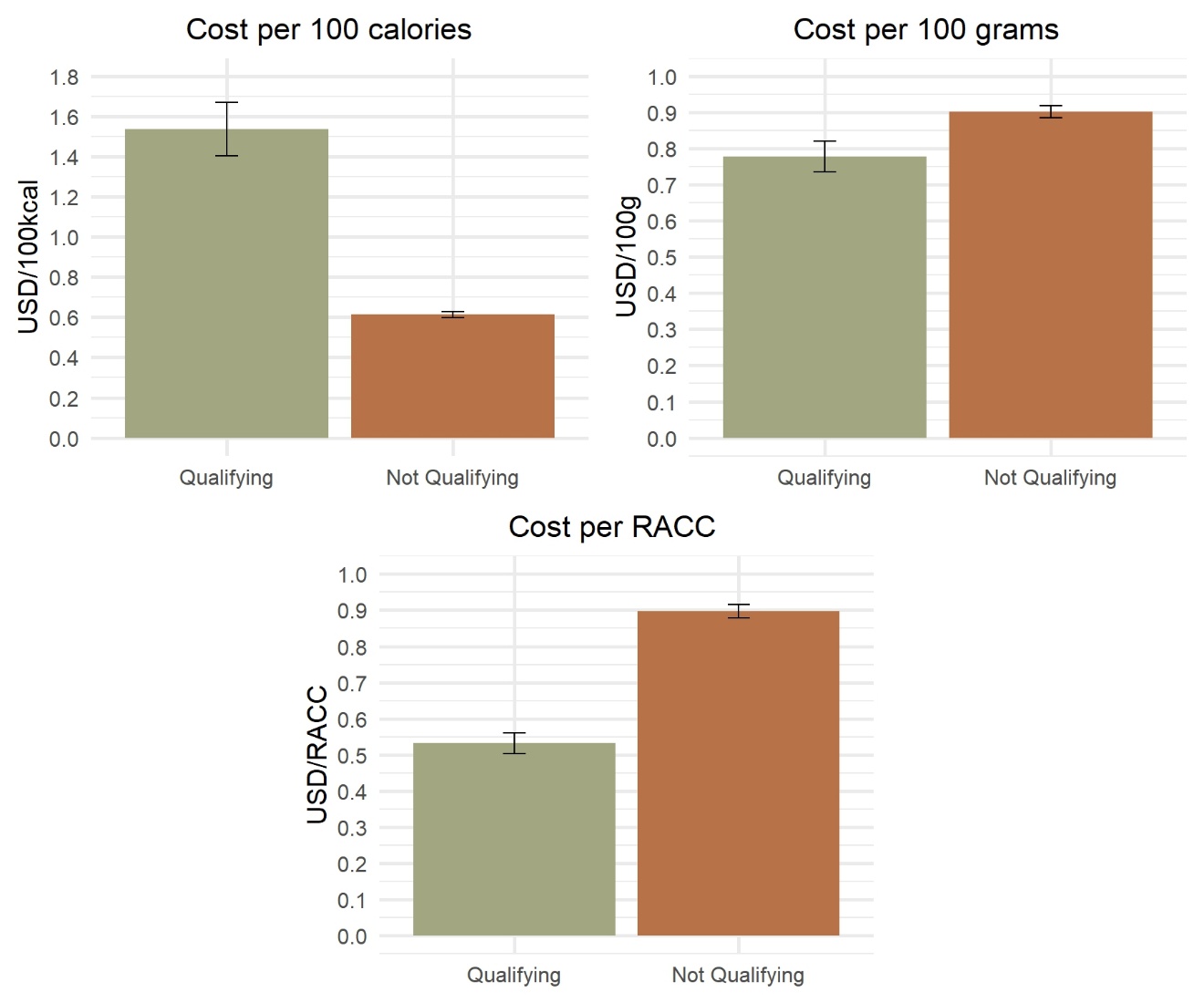


**Supplemental Figure 7.** Mean cost for Qualifying and Not Qualifying foods and beverages. Data represent mean costs calculated per 100 kilocalories, 100 grams, and per serving, determined by Reference Amounts Customarily Consumed (RACC). Error bars represent the standard error of the mean for each indicator. Foods and beverages are separated by whether they qualify under the new “healthy” definition criteria. Qualifying foods and beverages are those that meet the new criteria for the “healthy” definition, while Not Qualifying foods and beverages are those that do not.

Sensitivity Analysis Using Nutrients to Limit Criteria

For the 1178 foods and beverages not included in the main analysis, the strictest (2% MRV added sugar, 10% MRV sodium, 5% MRV saturated fat) and most lenient (20% MRV added sugar, 30% MRV sodium, 20% MRV saturated fat) criteria for the three nutrients to limit (NTL), sodium, added sugar, and saturated fat, were applied to determine the effects this excluded items might have on the nutrient density, cost, and frequency of consumption indicators.

Eight food groups were determined from the 1178 items. Mixed Dishes accounted for 432 items (37%), 321 were Desserts (27%), 176 (15%) were Snacks, 132 (11%) were Sauces and Condiments, 104 (9%) were Beverages, 5 (<1%) were Protein and Nutritional Powder, 5 (<1%) were Powdered Beverages, and 3 (<1%) were Oils. When the strict NTL criteria were applied, 189 (16%) of foods and beverages passed, and with the lenient NTL criteria applied, 652 (55%) of foods and beverages passed. **Supplemental Table 3** shows the distribution of foods and beverages by food group and whether they passed the strict criteria; **Supplemental Table 4** shows the distribution for the lenient criteria. Far more items failed the strict criteria than passed among every food group, while the lenient criteria resulted in more items passing than failing among Mixed Dishes, Snacks, and Protein and Nutritional Powder. Of items that passed the strict criteria, 65% were Mixed Dishes, 15% were Beverages, and 11% were Sauces and Condiments. Mixed Dishes also accounted for the largest proportion of items that passed the lenient criteria (60%), followed by Snacks at 19%, and Sauces and Condiments with 10%.

**Supplemental Table 3**. Distribution of items across food groups and whether they passed the strict^1^ Nutrients to Limit (NTL) criteria. Foods and beverages that passed satisfy all three criteria for added sugar, sodium, and saturated fat; those that failed exceeded the limits of at least one criterion.

| Food Group | Overall | Passed the strict NTL criteria | |
| --- | --- | --- | --- |
|  |  | Yes | No |
|  | n (% of Total) | n (% of Food Group) | n (% of Food Group) |
| Total | 1178 | 189 | 989 |
|  | 100.00% | 16.04% | 83.96% |
| Beverages | 104 | 29 | 75 |
|  | 8.83% | 27.88% | 72.12% |
| Desserts | 321 | 4 | 317 |
|  | 27.25% | 1.25% | 98.75% |
| Mixed Dishes | 432 | 123 | 309 |
|  | 36.67% | 28.47% | 71.53% |
| Oils | 3 | 0 | 3 |
|  | 0.25% | -- | 100.00% |
| Powdered Beverages | 5 | 1 | 4 |
|  | 0.42% | 20.00% | 80.00% |
| Protein and Nutritional Powder | 5 | 1 | 4 |
|  | 0.42% | 20.00% | 80.00% |
| Sauces and Condiments | 132 | 21 | 111 |
|  | 11.21% | 15.91% | 84.09% |
| Snacks | 176 | 10 | 166 |
|  | 14.94% | 5.68% | 94.32% |

^1^Strict NTL criteria: foods and beverages must not contain more than 2% MRV of added sugar, 10% MRV of sodium, and 5% MRV of saturated fat.

**Supplemental Table 4**. Distribution of items across food groups and whether they passed the lenient^1^ Nutrients to Limit (NTL) criteria. Foods and beverages that passed satisfy all three criteria for added sugar, sodium, and saturated fat; those that failed exceeded the limits of at least one criterion.

| Food Group | Overall | Passed the lenient NTL criteria | |
| --- | --- | --- | --- |
|  |  | Yes | No |
|  | n (% of Total) | n (% of Food Group) | n (% of Food Group) |
| Total | 1178 | 652 | 526 |
|  | 100.00% | 55.35% | 44.65% |
| Beverages | 104 | 46 | 58 |
|  | 8.83% | 44.23% | 55.77% |
| Desserts | 321 | 24 | 297 |
|  | 27.25% | 7.48% | 92.52% |
| Mixed Dishes | 432 | 389 | 43 |
|  | 36.67% | 90.05% | 9.95% |
| Oils | 3 | 0 | 3 |
|  | 0.25% | -- | 100.00% |
| Powdered Beverages | 5 | 2 | 3 |
|  | 0.42% | 40.00% | 60.00% |
| Protein and Nutritional Powder | 5 | 3 | 2 |
|  | 0.42% | 60.00% | 40.00% |
| Sauces and Condiments | 132 | 63 | 69 |
|  | 11.21% | 47.73% | 52.27% |
| Snacks | 176 | 125 | 51 |
|  | 14.94% | 71.02% | 28.98% |

^1^Lenient NTL criteria: foods and beverages must not contain more than 20% MRV of added sugar, 30% MRV of sodium, and 20% MRV of saturated fat.

*Passed/Failed Strict and Lenient nutrients to limit criteria*

Of the 1178 items in this sensitivity analysis, 989 (84%) failed to meet the strict NTL criteria and 526 (45%) failed to meet the lenient NTL criteria. Failure to meet the criteria could be due to surpassing the limits on any one of three nutrients to limit: 1) added sugars; 2) saturated fats; or 3) sodium. Items could be disqualified for more than one reason, and the distribution of the numbers of items that passed or failed each criteria are shown in **Supplemental Figure 8**.

It should be noted that the total number of items that passed the lenient criteria also includes all items that passed the strict criteria, and the items shown to have passed the lenient criteria in blue refer only to items that passed the lenient criteria but not also the strict criteria.

When the strict NTL criteria were applied, the largest proportion of items exceeded the saturated fat limit of 5% MRV (60% of items). A similar number of items exceeded the sugar (46%) and sodium (47%) limits, which were 5% MRV and 10% MRV, respectively. With the lenient NTL criteria, 35% of items exceeded the limit for added sugar, which was 20% MRV. The limit of 20% MRV on saturated fat was exceeded by 19% of items, while only 3% of items exceeded the 30% MRV limit on sodium. Altogether, 84% of items failed one or more of the strict NTL criteria and 45% failed one or more of the lenient criteria.

The primary reason for failing the strict and lenient criteria also varied by food group, shown in **Supplemental Figure 9** and **Supplemental Figure 10**. When the strict criteria were applied, Beverages and Desserts primarily failed the added sugar criterion (65% and 95% of each food group, respectively); Protein and Nutritional Powder (80%) and Sauces and Condiments (54%) mostly failed the sodium criterion, and Mixed Dishes (56%), Oils (100%), and Snacks (70%) primarily failed the saturated fat criterion.

The lenient criteria application resulted in the added sugar criterion being the one most often failed, with the largest proportion of all but two food groups, Mixed Dishes and Oils, containing excess added sugar (56% of Beverages, 88% of Desserts, 40% each of Powdered Beverages and Protein and Nutritional Powder, 29% of Sauces and Condiments, and 19% of Snacks). Mixed Dishes and Oils both had more items fail the saturated fat criterion than the other criteria (6% and 100%, respectively).


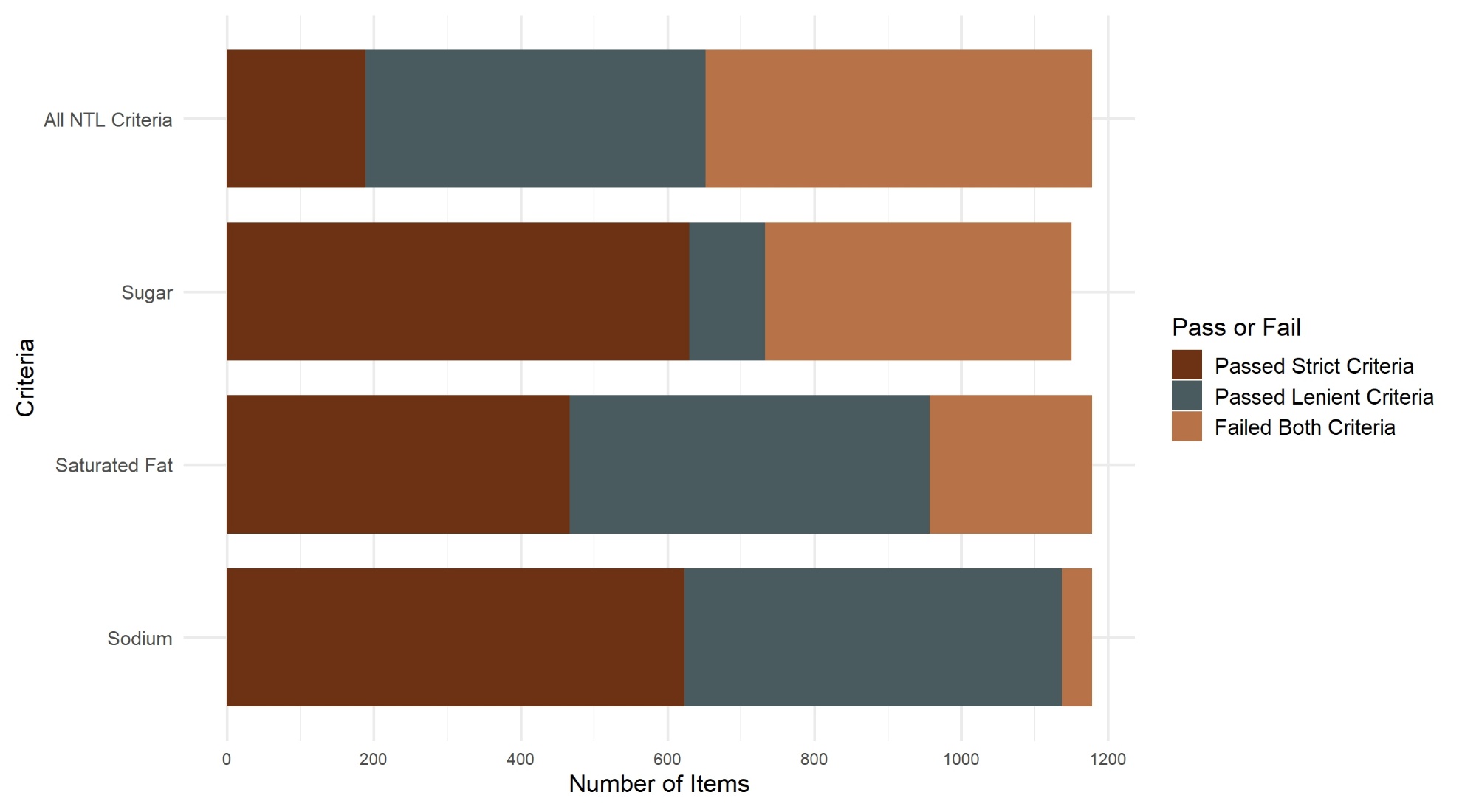


**Supplemental Figure 8:** Number of foods and beverages passing or failing the application of strict and lenient nutrients to limit (NTL) criteria. Bars represent, for items not included in the main analysis, the number of items that passed or failed each of the criteria for added sugar, sodium, and saturated fat individually, and overall. The number of items that passed the lenient criteria (shown in blue) only accounts for items that did not also pass the strict criteria; therefore, the total number of items passing the lenient criteria also includes those that passed the criteria.


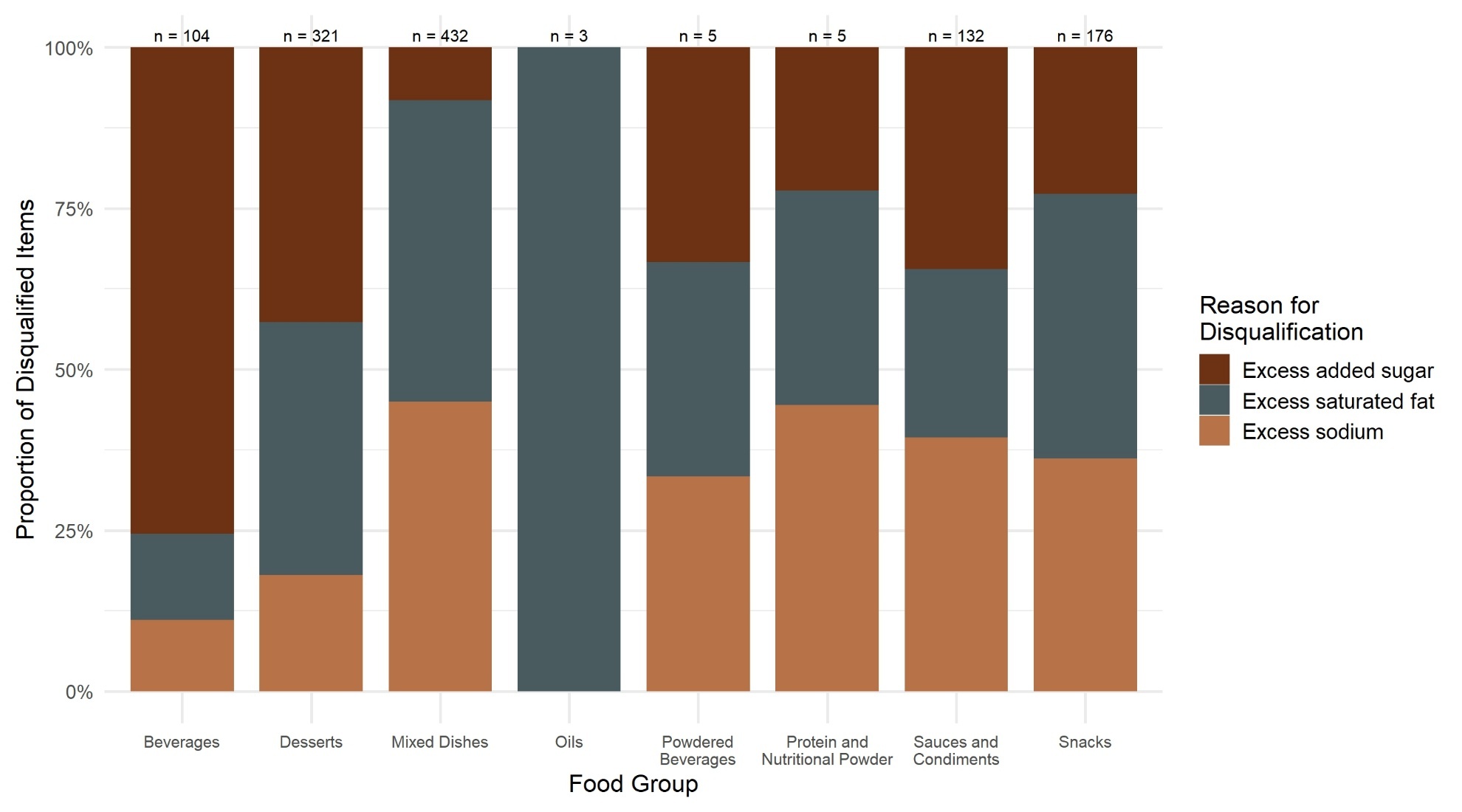


**Supplemental Figure 9:** Proportion of reasons for failing the application of strict nutrients to limit (NTL) criteria by food group. Bars represent, for items not included in the main analysis and that failed the strict NTL criteria, the proportion of items that were disqualified for each of three reasons. Foods and beverages could be disqualified for any combination of the possible reasons; therefore, some items may be counted more than once for each food group if they were disqualified for more than one reason. N represents the total number of items in the respective food group, not the total count of each bar.


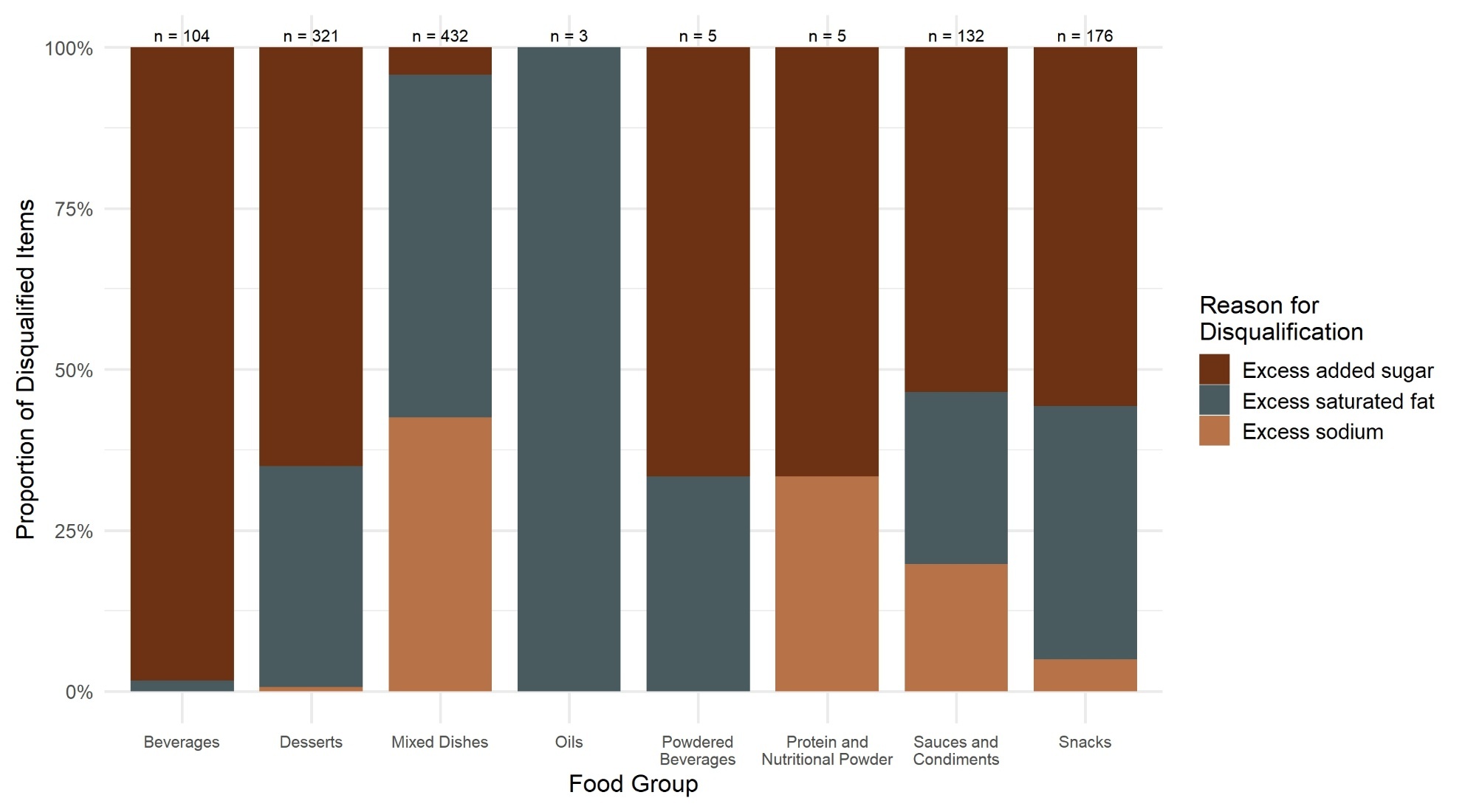


**Supplemental Figure 10:** Proportion of reasons for failing the application of lenient nutrients to limit (NTL) criteria by food group. Bars represent, for items not included in the main analysis and that failed the lenient NTL criteria, the proportion of items that were disqualified for each of three reasons. Foods and beverages could be disqualified for any combination of the possible reasons; therefore, some items may be counted more than once for each food group if they were disqualified for more than one reason. N represents the total number of items in the respective food group, not the total count of each bar.

Nutrient density, cost, and acceptability of Passed/Failed foods

**Supplemental Table 5** displays the medians and interquartile ranges for nutrient density, monetary cost, and frequency of consumption, overall and by food group for the application of the strict NTL criteria to the 1178 items not included in the main analysis. **Supplemental Table 6** shows the same metrics for the application of the lenient NTL criteria. Across all 1178 items, the median nutrient density score, based on the NRF 9.3, among all items was 0.08. The median cost per serving was $0.36, and the median weighted frequency of consumption was 0.34 occurrences per person-year. Powdered Beverages had the highest nutrient density score, while Sauces and Condiments had the lowest. Oils had both the lowest cost and frequency of consumption, and Protein and Nutritional Powders had the highest values for both indicators.

Shown in **Supplemental Figure 11** there were significant differences in the nutrient density, monetary cost, and frequency of consumption metrics between foods and beverages that passed and failed the strict criteria (p < 0.001 for nutrient density and cost; p < 0.01 for frequency), with items that passed having a higher median nutrient density and lower cost per serving and frequency of consumption than items that failed. Mean values for nutrition, cost, and frequency of consumption followed the same trend of items that passed the strict criteria being more nutrient-dense, cheaper, and less frequently consumed, shown in **Supplemental Figure 12**.

Items that passed the strict NTL criteria had a higher median nutrient density score than those that failed in every food group except Powdered Beverages (p < 0.001 for Beverages, Mixed Dishes, and Sauces and Condiments). All but two food groups, Powdered Beverages and Sauces and Condiments, also had lower costs among items that failed (p < 0.001 for Mixed Dishes; p < 0.05 for Desserts). Likewise, only Desserts and Sauces and Condiments had items that passed that were more frequently consumed than items that failed in the same food group.

Significant differences between foods and beverages that passed and failed the strict criteria were found only for nutrient density and cost per serving (p < 0.001 for both), with items that passed again having a higher median nutrient density and lower cost per serving, as well as a lower frequency of consumption (**Supplemental Figure 13**). Mean values for nutrition, cost, and frequency of consumption followed the same trend of items that passed the lenient criteria being more nutrient-dense, cheaper, and less frequently consumed, shown in **Supplemental Figure 14**.

Items that passed the lenient NTL criteria again had a higher median nutrient density score than those that failed in every food group except Powdered Beverages and Protein and Nutritional Powder (p < 0.001 for Beverages, Desserts, Mixed Dishes, and Sauces and Condiments; p < 0.05 for Snacks). All but two food groups, Mixed Dishes and Snacks, also had lower costs among items that failed (p < 0.001 for Mixed Dishes and Snacks). Food groups were nearly split for frequency of consumption, with Beverages, Desserts, and Powdered Beverages having a greater frequency of consumption among items that failed, and Mixed Dishes (p < 0.05), Protein and Nutritional Powder, Sauces and Condiments, and Snacks being consumed more often among items that passed the strict criteria.

**Supplemental Table 5**. Nutrient Density (NRF 9.3 score), cost (USD ($) per serving, quantified by RACC), and weighted frequency of consumption (per person-year) for the 1178 items not included in the main analysis. Metrics are shown across food groups and whether they passed the strict^1^ Nutrients to Limit (NTL) criteria. Data represent median values and interquartile range (IQR). Foods and beverages that passed satisfy all three criteria for added sugar, sodium, and saturated fat; those that failed exceeded the limits of at least one criterion. Statistically significant differences in indicator scores between items that passed and failed the NTL criteria assessed via Mann-Whitney U tests are indicated as: *** p-value < 0.001; ** p-value < 0.01; * p-value < 0.05.

| Food Group | Nutrient Density (NRF 9.3) | | | Cost (USD) per RACC | | | Weighted Frequency per Person-Year | | |  |
| --- | --- | --- | --- | --- | --- | --- | --- | --- | --- | --- |
|  | Overall | Passed | Failed | Overall | Passed | Failed | Overall | Passed | Failed | |
|  | Median [IQR] | | | Median [IQR] | | | Median [IQR] | | | |
| Total | 0.08 | 0.27*** | 0.06 | 0.36 | 0.14*** | 0.39 | 0.34 | 0.25** | 0.36 | |
|  | [-0.02-0.2] | [0.14-0.43] | [-0.03-0.15] | [0.19-0.64] | [0.07-0.36] | [0.23-0.72] | [0.1-0.98] | [0.07-0.65] | [0.11-1.05] | |
| Beverages | 0.31 | 1.19*** | 0.06 | 0.38 | 0.37 | 0.39 | 0.56 | 0.39 | 0.66 | |
|  | [-0.09-0.87] | [0.56-3.02] | [-0.11-0.67] | [0.33-1.01] | [0.33-0.57] | [0.31-1.13] | [0.16-2.1] | [0.18-1.14] | [0.15-2.47] | |
| Desserts | 0.00 | 0.02 | 0.00 | 0.59 | 0.28* | 0.60 | 0.30 | 0.45 | 0.30 | |
|  | [-0.05-0.05] | [0-0.19] | [-0.05-0.05] | [0.38-1.09] | [0.26-0.35] | [0.4-1.09] | [0.1-1.07] | [0.21-1.52] | [0.1-1.07] | |
| Mixed Dishes | 0.14 | 0.22*** | 0.12 | 0.27 | 0.11*** | 0.41 | 0.22 | 0.17 | 0.24 | |
|  | [0.08-0.23] | [0.12-0.33] | [0.07-0.19] | [0.14-0.57] | [0.06-0.2] | [0.22-0.63] | [0.07-0.59] | [0.05-0.51] | [0.08-0.61] | |
| Oils | 0.01 | -- | 0.01 | 0.06 | -- | 0.06 | 0.05 | -- | 0.05 | |
|  | [-0.03-0.02] | -- | [-0.03-0.02] | [0.04-0.18] | -- | [0.04-0.18] | [0.03-0.31] | -- | [0.03-0.31] | |
| Powdered Beverages | 0.80 | 0.60 | 1.01 | 0.17 | 0.47 | 0.16 | 0.26 | 0.26 | 0.35 | |
|  | [0.6-1.22] | [0.6-0.6] | [0.63-1.32] | [0.15-0.23] | [0.47-0.47] | [0.12-0.18] | [0.24-0.46] | [0.26-0.26] | [0.24-0.54] | |
| Protein and Nutritional Powders | 0.73 | 1.42 | 0.73 | 2.85 | 0.88 | 3.12 | 1.18 | 1.10 | 1.42 | |
|  | [0.73-1.09] | [1.42-1.42] | [0.68-0.82] | [1.62-3.4] | [1.35-1.35] | [1.35-1.35] | [1.1-1.67] | [1.1-1.1] | [0.98-1.7] | |
| Sauces and Condiments | -0.07 | 0.48*** | -0.08 | 0.17 | 0.14 | 0.20 | 0.52 | 0.30 | 0.55 | |
|  | [-0.12-0.09] | [0.3-0.66] | [-0.13-0.03] | [0.12-0.3] | [0.04-0.34] | [0.12-0.3] | [0.18-1.85] | [0.13-1.1] | [0.19-1.92] | |
| Snacks | 0.13 | 0.16 | 0.13 | 0.31 | 0.22 | 0.31 | 0.55 | 0.22 | 0.56 | |
|  | [0.05-0.2] | [0.14-0.19] | [0.05-0.21] | [0.19-0.41] | [0.2-0.32] | [0.18-0.42] | [0.2-1.27] | [0.07-0.47] | [0.21-1.31] | |

^1^Strict NTL criteria: foods and beverages must not contain more than 2% MRV of added sugar, 10% MRV of sodium, and 5% MRV of saturated fat.

**Supplemental Table 6**. Nutrient Density (NRF 9.3 score), cost (USD ($) per serving, quantified by RACC), and weighted frequency of consumption (per person-year) for the 1178 items not included in the main analysis. Metrics are shown across food groups and whether they passed the lenient^1^ Nutrients to Limit (NTL) criteria. Data represent median values and interquartile range (IQR). Foods and beverages that passed satisfy all three criteria for added sugar, sodium, and saturated fat; those that failed exceeded the limits of at least one criterion. Statistically significant differences in indicator scores between items that passed and failed the NTL criteria assessed via Mann-Whitney U tests are indicated as: *** p-value < 0.001; ** p-value < 0.01; * p-value < 0.05.

| Food Group | Nutrient Density (NRF 9.3) | | | Cost (USD) per RACC | | | Weighted Frequency per Person-Year | | |  |
| --- | --- | --- | --- | --- | --- | --- | --- | --- | --- | --- |
|  | Overall | Passed | Failed | Overall | Passed | Failed | Overall | Passed | Failed | |
|  | Median [IQR] | | | Median [IQR] | | | Median [IQR] | | | |
| Total | 0.08 | 0.14*** | -0.01 | 0.36 | 0.27*** | 0.46 | 0.34 | 0.32 | 0.36 | |
|  | [-0.02-0.2] | [0.07-0.25] | [-0.07-0.07] | [0.19-0.64] | [0.14-0.48] | [0.3-0.95] | [0.1-0.98] | [0.1-0.82] | [0.1-1.16] | |
| Beverages | 0.31 | 1.03*** | 0.04 | 0.38 | 0.41 | 0.37 | 0.56 | 0.47 | 0.71 | |
|  | [-0.09-0.87] | [0.33-2.02] | [-0.11-0.52] | [0.33-1.01] | [0.34-1.1] | [0.31-0.83] | [0.16-2.1] | [0.19-1.08] | [0.1-2.76] | |
| Desserts | 0.00 | 0.10*** | -0.01 | 0.59 | 0.62 | 0.58 | 0.30 | 0.13 | 0.32 | |
|  | [-0.05-0.05] | [0.04-0.18] | [-0.05-0.04] | [0.38-1.09] | [0.51-0.83] | [0.38-1.11] | [0.1-1.07] | [0.09-0.33] | [0.1-1.11] | |
| Mixed Dishes | 0.14 | 0.14*** | 0.08 | 0.27 | 0.25*** | 0.76 | 0.22 | 0.23* | 0.14 | |
|  | [0.08-0.23] | [0.09-0.24] | [-0.01-0.18] | [0.14-0.57] | [0.12-0.5] | [0.57-1.06] | [0.07-0.59] | [0.07-0.61] | [0.03-0.37] | |
| Oils | 0.01 | -- | 0.01 | 0.06 | -- | 0.06 | 0.05 | -- | 0.05 | |
|  | [-0.03-0.02] | -- | [-0.03-0.02] | [0.04-0.18] | -- | [0.04-0.18] | [0.03-0.31] | -- | [0.03-0.31] | |
| Powdered Beverages | 0.80 | 0.70 | 1.22 | 0.17 | 0.25 | 0.17 | 0.26 | 0.25 | 0.46 | |
|  | [0.6-1.22] | [0.65-0.75] | [0.67-1.42] | [0.15-0.23] | [0.14-0.36] | [0.16-0.2] | [0.24-0.46] | [0.25-0.26] | [0.34-0.62] | |
| Protein and Nutritional Powders | 0.73 | 0.73 | 0.81 | 2.85 | 2.85 | 2.78 | 1.18 | 1.67 | 0.79 | |
|  | [0.73-1.09] | [0.73-1.07] | [0.67-0.95] | [1.62-3.4] | [1.35-1.35] | [1.35-1.35] | [1.1-1.67] | [1.39-1.72] | [0.6-0.98] | |
| Sauces and Condiments | -0.07 | 0.07*** | -0.10 | 0.17 | 0.17 | 0.21 | 0.52 | 0.61 | 0.52 | |
|  | [-0.12-0.09] | [-0.09-0.32] | [-0.16--0.06] | [0.12-0.3] | [0.09-0.31] | [0.12-0.3] | [0.18-1.85] | [0.2-2.63] | [0.16-1.31] | |
| Snacks | 0.13 | 0.13* | 0.10 | 0.31 | 0.25*** | 0.38 | 0.55 | 0.56 | 0.44 | |
|  | [0.05-0.2] | [0.06-0.2] | [0-0.2] | [0.19-0.41] | [0.15-0.35] | [0.32-0.49] | [0.2-1.27] | [0.2-1.4] | [0.2-1.14] | |

^1^Lenient NTL criteria: foods and beverages must not contain more than 20% MRV of added sugar, 30% MRV of sodium, and 20% MRV of saturated fat.

**
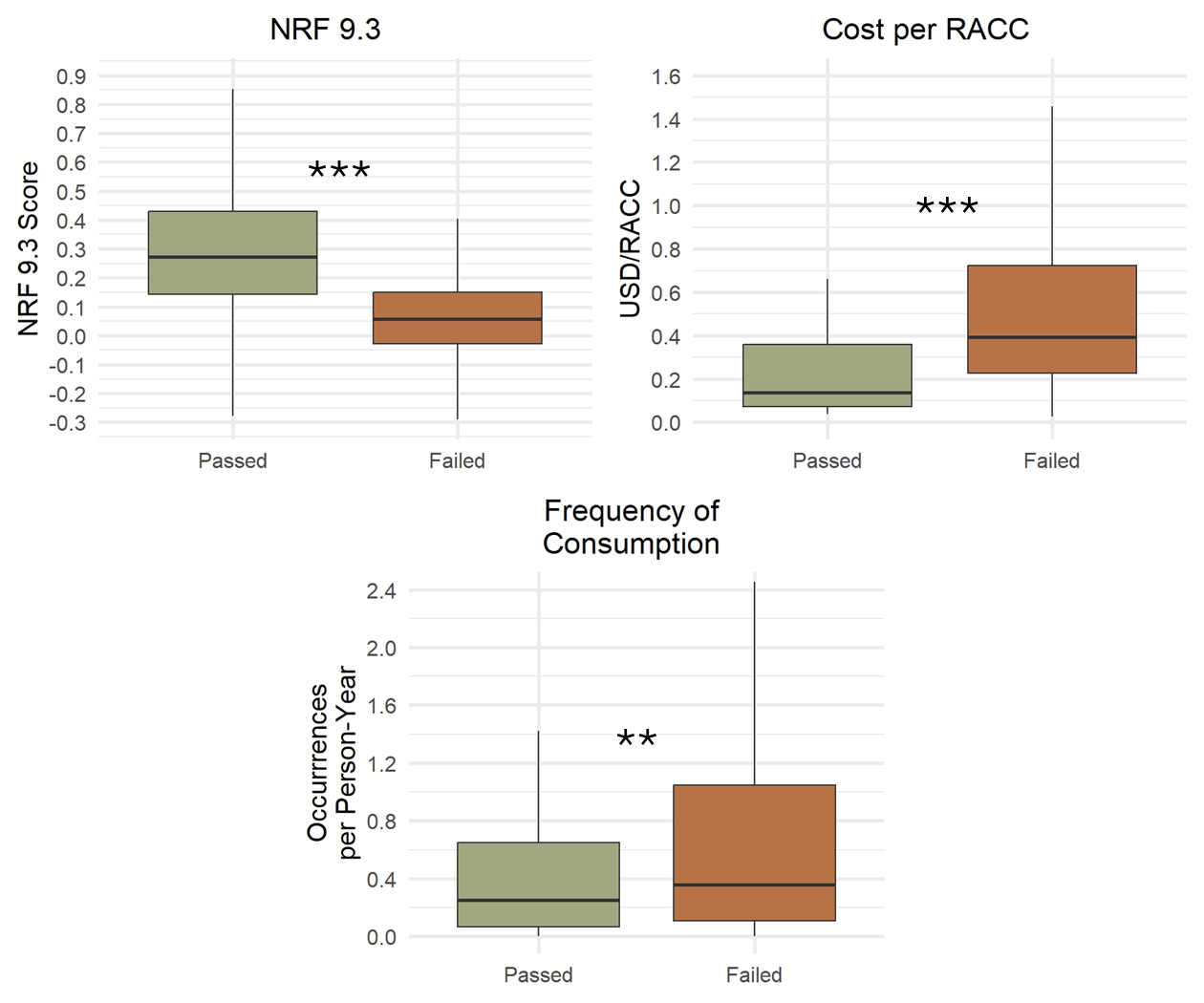
**

**Supplemental Figure 11:** Median indicator scores for the 1178 foods and beverages not included in the main analysis, resultant from the strict Nutrients to Limit (NTL) criteria application. Boxes represent, for items not included in the main analysis, median and interquartile range for nutrient density determined by NRF 9.3 scores, cost in USD per serving, and weighted frequency of consumption per person-year. Whiskers represent minimum and maximum for each indicator. Values exclude outliers, defined as exceeding 1.5 times the IQR. Foods and beverages are separated by whether they passed the strict NTL criteria. Foods and beverages that passed satisfy all three criteria for added sugar, sodium, and saturated fat, while those that failed exceeded the limits of at least one criterion. Statistically significant differences between Qualifying and Not Qualifying items assessed via Mann-Whitney U-tests are indicated as: *** p-value < 0.001; ** p-value < 0.01. The number of outliers for each indicator are as follows: NRF 9.3: Passed = 3 lower/17 upper, Failed= 22 lower/54 upper; Cost: Passed = 21 upper, Failed = 62 upper; Frequency: Passed = 16 upper, Failed = 103 upper.

**
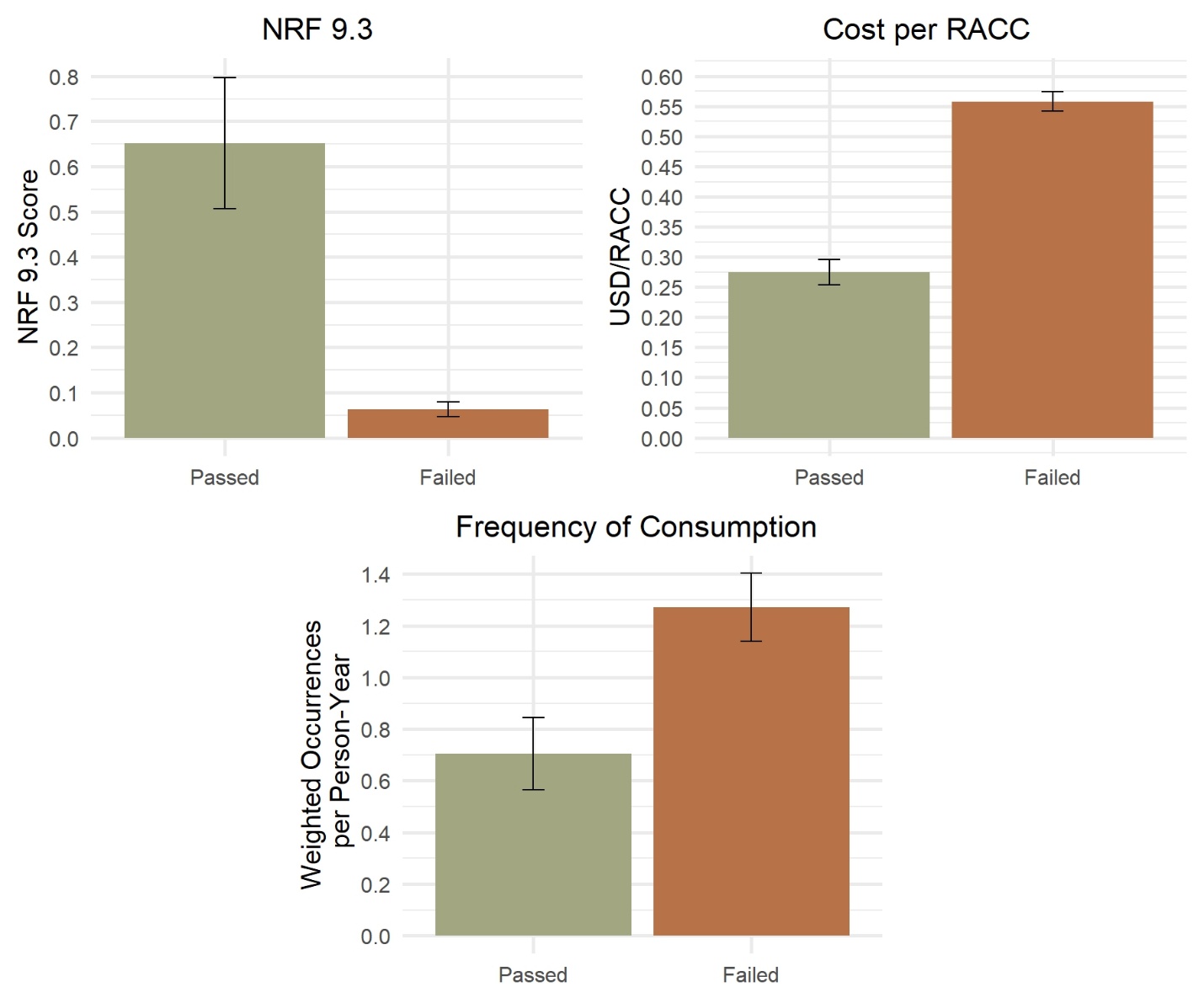
**

**Supplemental Figure 12**. Mean indicator scores for the 1178 foods and beverages not included in the main analysis, resultant from the strict Nutrients to Limit (NTL) criteria application. Data represent, for items not included in the main analysis, mean nutrient density determined by NRF 9.3 scores, cost in USD per serving, and weighted frequency of consumption per person-year. Error bars represent the standard error of the mean for each indicator. Foods and beverages are separated by whether they passed the strict NTL criteria. Foods and beverages that passed satisfy all three criteria for added sugar, sodium, and saturated fat, while those that failed exceeded the limits of at least one criterion.

**
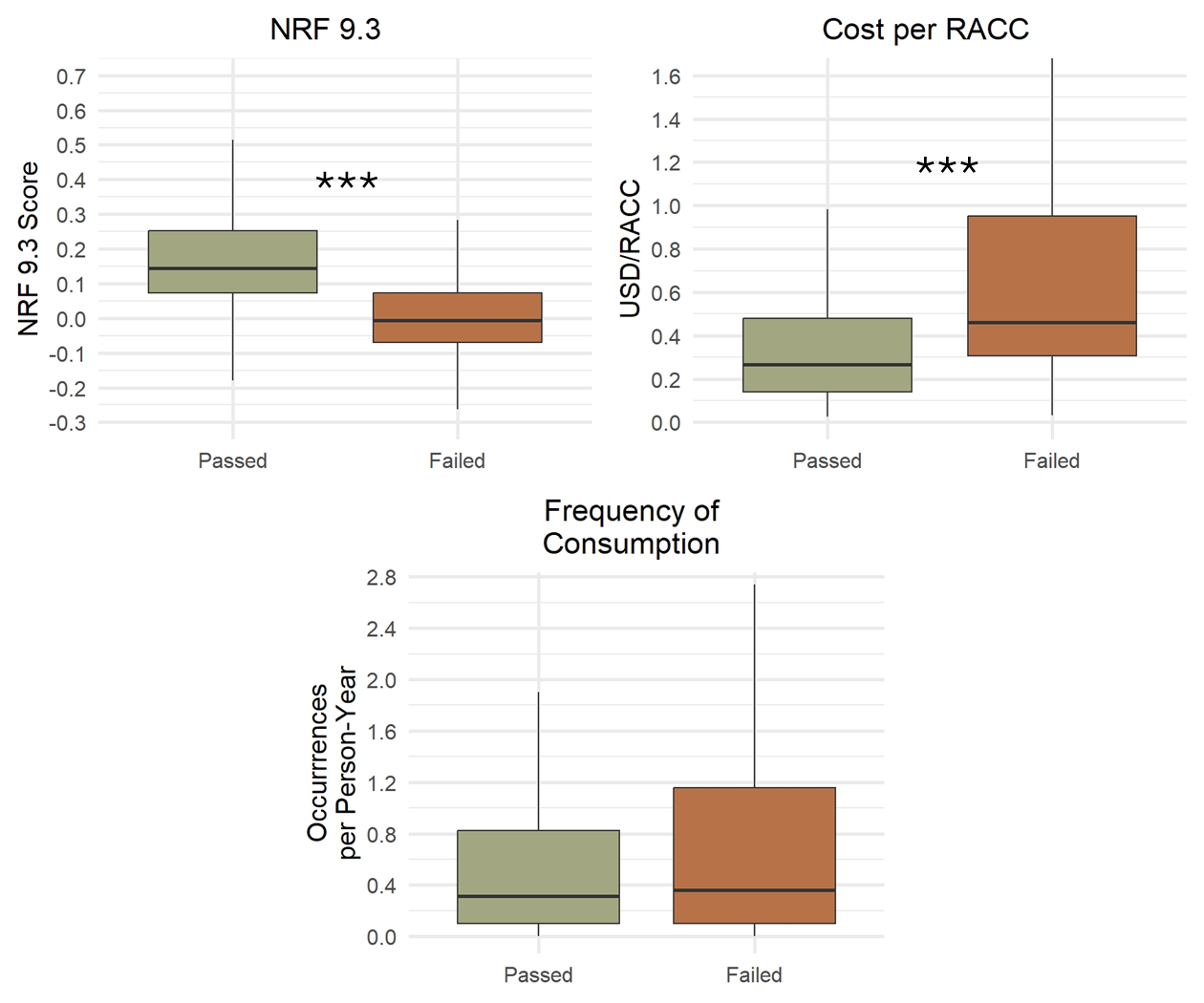
**

**Supplemental Figure 13:** Median indicator scores for the 1178 foods and beverages not included in the main analysis, resultant from the lenient Nutrients to Limit (NTL) criteria application. Boxes represent, for items not included in the main analysis, median and interquartile range for nutrient density determined by NRF 9.3 scores, cost in USD per serving, and weighted frequency of consumption per person-year. Whiskers represent minimum and maximum for each indicator. Values exclude outliers, defined as exceeding 1.5 times the IQR. Foods and beverages are separated by whether they passed the lenient NTL criteria. Foods and beverages that passed satisfy all three criteria for added sugar, sodium, and saturated fat, while those that failed exceeded the limits of at least one criterion. Statistically significant differences between Qualifying and Not Qualifying items assessed via Mann-Whitney U-tests are indicated as: *** p-value < 0.001; ** p-value < 0.01. The number of outliers for each indicator are as follows: NRF 9.3: Passed = 3 lower/17 upper, Failed= 22 lower/54 upper; Cost: Passed = 21 upper, Failed = 62 upper; Frequency: Passed = 16 upper, Failed = 103 upper

**
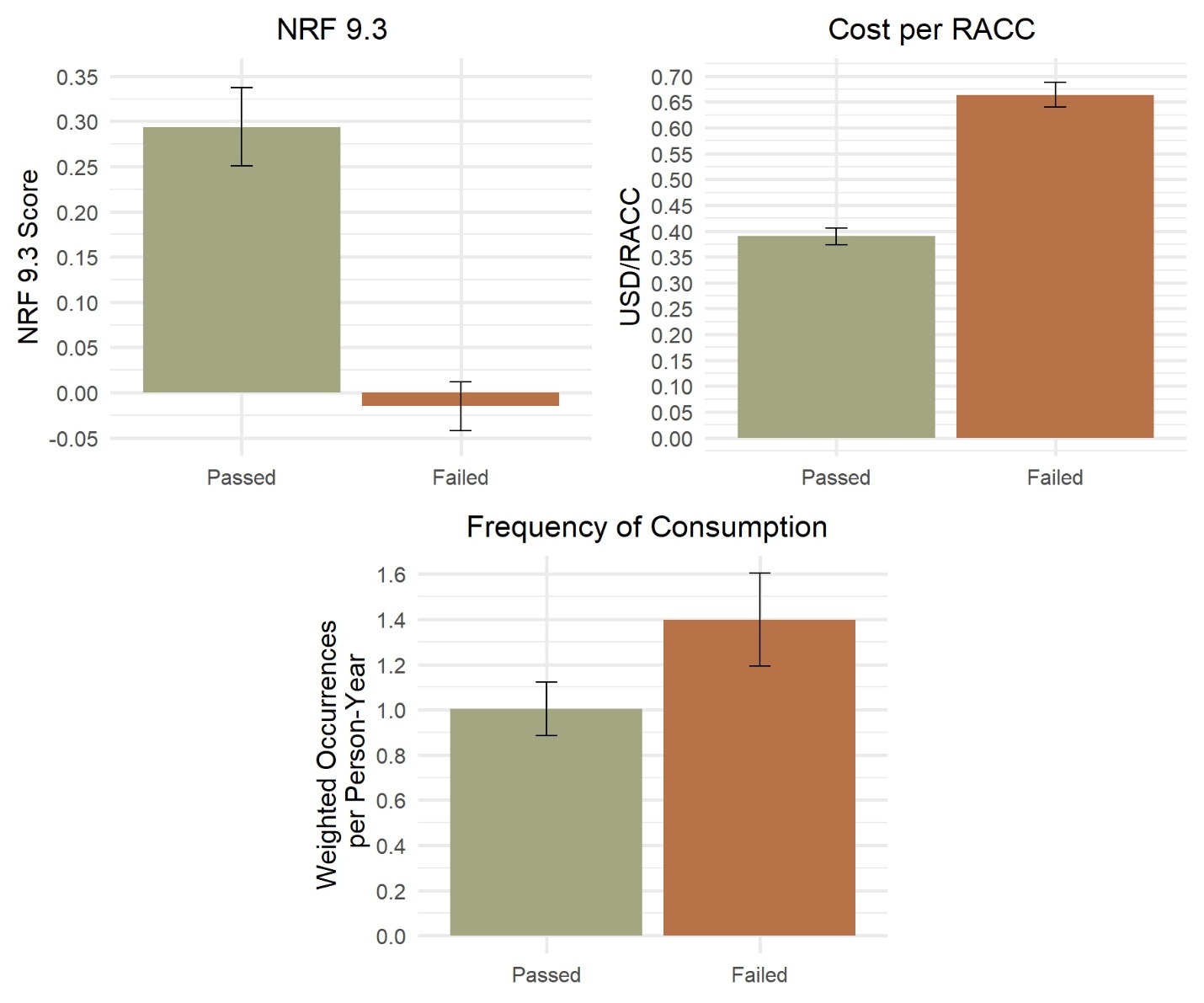
**

**Supplemental Figure 14:** Mean indicator scores for the 1178 foods and beverages not included in the main analysis, resultant from the lenient Nutrients to Limit (NTL) criteria application. Data represent, for items not included in the main analysis, mean nutrient density determined by NRF 9.3 scores, cost in USD per serving, and weighted frequency of consumption per person-year. Error bars represent the standard error of the mean for each indicator. Foods and beverages are separated by whether they passed the lenient NTL criteria. Foods and beverages that passed satisfy all three criteria for added sugar, sodium, and saturated fat, while those that failed exceeded the limits of at least one criterion.

Cost of Passed/Failed foods

**Supplemental Table 7** displays the medians and interquartile ranges for cost calculated per 100 kilocalories, per 100 grams, and per serving, as quantified by RACC, overall and by food group for the application of the strict NTL criteria to the 1178 items not included in the main analysis. **Supplemental Table 8** shows the same three cost metrics for the application of the lenient NTL criteria. Across all 1178 items, the median cost per 100 kilocalories was $0.30. The median cost per 100 grams was $0.76, and the median cost per serving was $0.36. Oils had the lowest cost per 100 kilocalories and per serving. Protein and Nutritional Powder had the highest cost per 100 grams and per serving. Beverages had the highest cost per 100 kilocalories but the lowest cost per 100 grams.

Also shown in **Supplemental Table 7**, there were significant differences in the costs calculated by different functional units between foods and beverages that passed and failed the strict criteria (p < 0.001 for all), with items that passed the strict criteria having a greater cost per 100 kilocalories and lower costs per 100 grams and per serving. Mean values followed the same trends for cost per 100 kilocalories, per 100 grams, and per serving, with items that passed being more expensive per 100 kilocalories but cheaper per 100 grams and per serving, shown in **Supplemental Figure 15**.

By food group, items that passed the strict NTL criteria also tended to be more expensive per 100 kilocalories than items that failed the strict criteria. Only among Mixed Dishes and Protein and Nutritional Powder were items that failed more expensive per 100 kilocalories (p < 0.001 for Beverages, Desserts, and Sauces and Condiments; p < 0.05 for Mixed Dishes). Although overall, costs per 100 grams, were cheaper for items that passed the strict criteria, the same was true only among Mixed Dishes (p < 0.001), while the other food groups had lower costs for the items that failed. When cost was calculated per serving, only Powdered Beverages and Sauces and Condiments were more expensive for items that passed the strict criteria (p < 0.001 for Mixed Dishes; p < 0.05 for Desserts).

When the lenient NTL criteria were applied, the same significant differences were found where foods and beverages that passed the criteria were more expensive per 100 kilocalories but cheaper per 100 grams and per serving compared to items that failed the criteria (**Supplemental Table 8**; p < 0.001 for all). This was again replicated in the mean values for the three cost metrics (**Supplemental Figure 16**).

Items that passed the lenient NTL criteria also had a higher cost per 100 kilocalories within every food group except Mixed Dishes and Snacks, where items that failed were more expensive (p < 0.001 for Beverages, Desserts, and Sauces and Condiments). With costs calculated per 100 grams, food groups were nearly split between foods that passed being cheaper or more expensive. Items that passed belonging to Beverages, Powdered Beverages, and Sauces and Condiments were more expensive per 100 grams, while items that passed in Desserts, Mixed Dishes (p < 0.01), and Snacks were cheaper. Finally, all but two food groups, Mixed Dishes and Snacks (p < 0.001 for both), had lower costs per serving among items that failed.

**Supplemental Table 7**. Cost (USD) calculated per 100 kilocalories, per 100 grams, and per serving, quantified by RACC for the 1178 items not included in the main analysis. Metrics are shown across food groups and whether they passed the strict^1^ Nutrients to Limit (NTL) criteria. Data represent median values and interquartile range (IQR). Foods and beverages that passed satisfy all three criteria for added sugar, sodium, and saturated fat; those that failed exceeded the limits of at least one criterion. Statistically significant differences in indicator scores between Qualifying and Not Qualifying items assessed via Mann-Whitney U tests are indicated as: *** p-value < 0.001; ** p-value < 0.01; * p-value < 0.05.

| Food Group | Cost (USD) per 100kcal | | | Cost (USD) per 100g | | | Cost (USD) per RACC | | |  |
| --- | --- | --- | --- | --- | --- | --- | --- | --- | --- | --- |
|  | Overall | Passed | Failed | Overall | Passed | Failed | Overall | Passed | Failed | |
|  | Median [IQR] | | | Median [IQR] | | | Median [IQR] | | | |
| Total | 0.30 | 0.47*** | 0.29 | 0.76 | 0.30*** | 0.80 | 0.36 | 0.14*** | 0.39 | |
|  | [0.21-0.5] | [0.2-0.96] | [0.21-0.44] | [0.39-1.08] | [0.2-0.64] | [0.51-1.12] | [0.19-0.64] | [0.07-0.36] | [0.23-0.72] | |
| Beverages | 0.48 | 3.90*** | 0.32 | 0.15 | 0.16 | 0.14 | 0.38 | 0.37 | 0.39 | |
|  | [0.26-0.98] | [0.93-5.73] | [0.23-0.73] | [0.1-0.34] | [0.1-0.2] | [0.11-0.36] | [0.33-1.01] | [0.33-0.57] | [0.31-1.13] | |
| Desserts | 0.26 | 0.97*** | 0.26 | 0.97 | 1.44 | 0.97 | 0.59 | 0.28* | 0.60 | |
|  | [0.21-0.35] | [0.79-1.35] | [0.21-0.35] | [0.76-1.26] | [0.37-2.58] | [0.76-1.26] | [0.38-1.09] | [0.26-0.35] | [0.4-1.09] | |
| Mixed Dishes | 0.32 | 0.28* | 0.34 | 0.52 | 0.29*** | 0.62 | 0.27 | 0.11*** | 0.41 | |
|  | [0.24-0.64] | [0.18-0.69] | [0.26-0.63] | [0.3-0.81] | [0.22-0.49] | [0.38-0.81] | [0.14-0.57] | [0.06-0.2] | [0.22-0.63] | |
| Oils | 0.05 | -- | 0.05 | 0.42 | -- | 0.42 | 0.06 | -- | 0.06 | |
|  | [0.04-0.15] | -- | [0.04-0.15] | [0.33-1.33] | -- | [0.33-1.33] | [0.04-0.18] | -- | [0.04-0.18] | |
| Powdered Beverages | 0.30 | 0.88 | 0.27 | 1.14 | 3.11 | 1.05 | 0.17 | 0.47 | 0.16 | |
|  | [0.24-0.66] | [0.88-0.88] | [0.2-0.4] | [0.98-1.51] | [3.11-3.11] | [0.78-1.23] | [0.15-0.23] | [0.47-0.47] | [0.12-0.18] | |
| Protein and Nutritional Powders | 0.38 | 0.34 | 0.38 | 1.35 | 1.35 | 1.35 | 2.85 | 0.88 | 3.12 | |
|  | [0.35-0.38] | [1.42-1.42] | [0.68-0.82] | [1.35-1.35] | [0.34-0.34] | [0.37-0.38] | [1.62-3.4] | [1.35-1.35] | [1.35-1.35] | |
| Sauces and Condiments | 0.44 | 0.94*** | 0.42 | 0.70 | 0.52 | 0.72 | 0.17 | 0.14 | 0.20 | |
|  | [0.24-0.9] | [0.5-1.83] | [0.21-0.82] | [0.46-1.05] | [0.3-1.14] | [0.49-0.99] | [0.12-0.3] | [0.04-0.34] | [0.12-0.3] | |
| Snacks | 0.22 | 0.33 | 0.22 | 1.05 | 1.32 | 1.05 | 0.31 | 0.22 | 0.31 | |
|  | [0.19-0.31] | [0.19-0.43] | [0.19-0.31] | [0.85-1.39] | [0.67-1.66] | [0.86-1.33] | [0.19-0.41] | [0.2-0.32] | [0.18-0.42] | |

^1^Strict NTL criteria: foods and beverages must not contain more than 2% MRV of added sugar, 10% MRV of sodium, and 5% MRV of saturated fat.


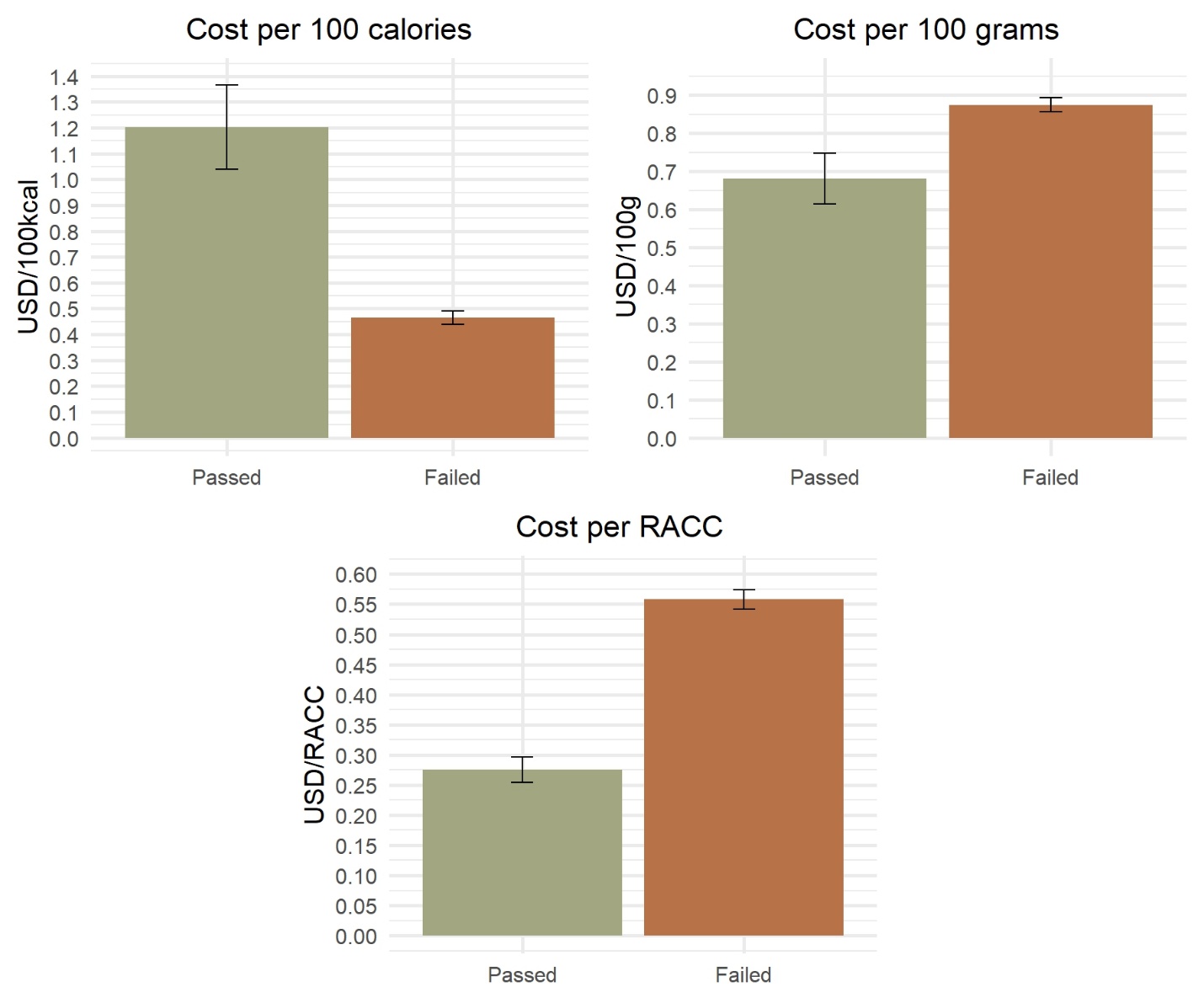


**Supplemental Figure 15**: Mean cost for the 1178 foods and beverages not included in the main analysis, resultant from the strict Nutrients to Limit (NTL) criteria application. Data represent mean costs calculated per 100 kilocalories, 100 grams, and per serving, determined by Reference Amounts Customarily Consumed (RACC). Error bars represent the standard error of the mean for each indicator. Foods and beverages are separated by whether they passed the strict NTL criteria. Foods and beverages that passed satisfied all three criteria for added sugar, sodium, and saturated fat, while those that failed exceeded the limits of at least one criterion.

**Supplemental Table 8**. Cost (USD) calculated per 100 kilocalories, per 100 grams, and per serving, quantified by RACC for the 1178 items not included in the main analysis. Metrics are shown across food groups and whether they passed the lenient^1^ Nutrients to Limit (NTL) criteria. Data represent median values and interquartile range (IQR). Foods and beverages that passed satisfy all three criteria for added sugar, sodium, and saturated fat; those that failed exceeded the limits of at least one criterion. Statistically significant differences in indicator scores between Qualifying and Not Qualifying items assessed via Mann-Whitney U tests are indicated as: *** p-value < 0.001; ** p-value < 0.01; * p-value < 0.05.

| Food Group | Cost (USD) per 100kcal | | | Cost (USD) per 100g | | | Cost (USD) per RACC | | |  |
| --- | --- | --- | --- | --- | --- | --- | --- | --- | --- | --- |
|  | Overall | Passed | Failed | Overall | Passed | Failed | Overall | Passed | Failed | |
|  | Median [IQR] | | | Median [IQR] | | | Median [IQR] | | | |
| Total | 0.30 | 0.34*** | 0.26 | 0.76 | 0.63*** | 0.88 | 0.36 | 0.27*** | 0.46 | |
|  | [0.21-0.5] | [0.22-0.69] | [0.2-0.37] | [0.39-1.08] | [0.33-0.97] | [0.58-1.23] | [0.19-0.64] | [0.14-0.48] | [0.3-0.95] | |
| Beverages | 0.48 | 1.40*** | 0.29 | 0.15 | 0.17 | 0.13 | 0.38 | 0.41 | 0.37 | |
|  | [0.26-0.98] | [0.79-5.08] | [0.22-0.44] | [0.1-0.34] | [0.1-0.38] | [0.1-0.31] | [0.33-1.01] | [0.34-1.1] | [0.31-0.83] | |
| Desserts | 0.26 | 0.44*** | 0.26 | 0.97 | 0.93 | 0.97 | 0.59 | 0.62 | 0.58 | |
|  | [0.21-0.35] | [0.31-0.78] | [0.2-0.34] | [0.76-1.26] | [0.51-1.46] | [0.77-1.26] | [0.38-1.09] | [0.51-0.83] | [0.38-1.11] | |
| Mixed Dishes | 0.32 | 0.32 | 0.37 | 0.52 | 0.51** | 0.70 | 0.27 | 0.25*** | 0.76 | |
|  | [0.24-0.64] | [0.23-0.64] | [0.26-0.65] | [0.3-0.81] | [0.3-0.8] | [0.43-0.84] | [0.14-0.57] | [0.12-0.5] | [0.57-1.06] | |
| Oils | 0.05 | -- | 0.05 | 0.42 | -- | 0.42 | 0.06 | -- | 0.06 | |
|  | [0.04-0.15] | -- | [0.04-0.15] | [0.33-1.33] | -- | [0.33-1.33] | [0.04-0.18] | -- | [0.04-0.18] | |
| Powdered Beverages | 0.30 | 0.47 | 0.30 | 1.14 | 1.64 | 1.14 | 0.17 | 0.25 | 0.17 | |
|  | [0.24-0.66] | [0.26-0.67] | [0.27-0.48] | [0.98-1.51] | [0.91-2.38] | [1.05-1.32] | [0.15-0.23] | [0.14-0.36] | [0.16-0.2] | |
| Protein and Nutritional Powders | 0.38 | 0.38 | 0.36 | 1.35 | 1.35 | 1.35 | 2.85 | 2.85 | 2.78 | |
|  | [0.35-0.38] | [0.73-1.07] | [0.67-0.95] | [1.35-1.35] | [0.36-0.38] | [0.36-0.37] | [1.62-3.4] | [1.35-1.35] | [1.35-1.35] | |
| Sauces and Condiments | 0.44 | 0.70*** | 0.25 | 0.70 | 0.69 | 0.72 | 0.17 | 0.17 | 0.21 | |
|  | [0.24-0.9] | [0.46-1.11] | [0.16-0.45] | [0.46-1.05] | [0.46-1.07] | [0.48-0.99] | [0.12-0.3] | [0.09-0.31] | [0.12-0.3] | |
| Snacks | 0.22 | 0.22 | 0.26 | 1.05 | 1.03 | 1.12 | 0.31 | 0.25*** | 0.38 | |
|  | [0.19-0.31] | [0.18-0.31] | [0.21-0.32] | [0.85-1.39] | [0.8-1.42] | [0.93-1.34] | [0.19-0.41] | [0.15-0.35] | [0.32-0.49] | |

^1^Lenient NTL criteria: foods and beverages must not contain more than 20% MRV of added sugar, 30% MRV of sodium, and 20% MRV of saturated fat.


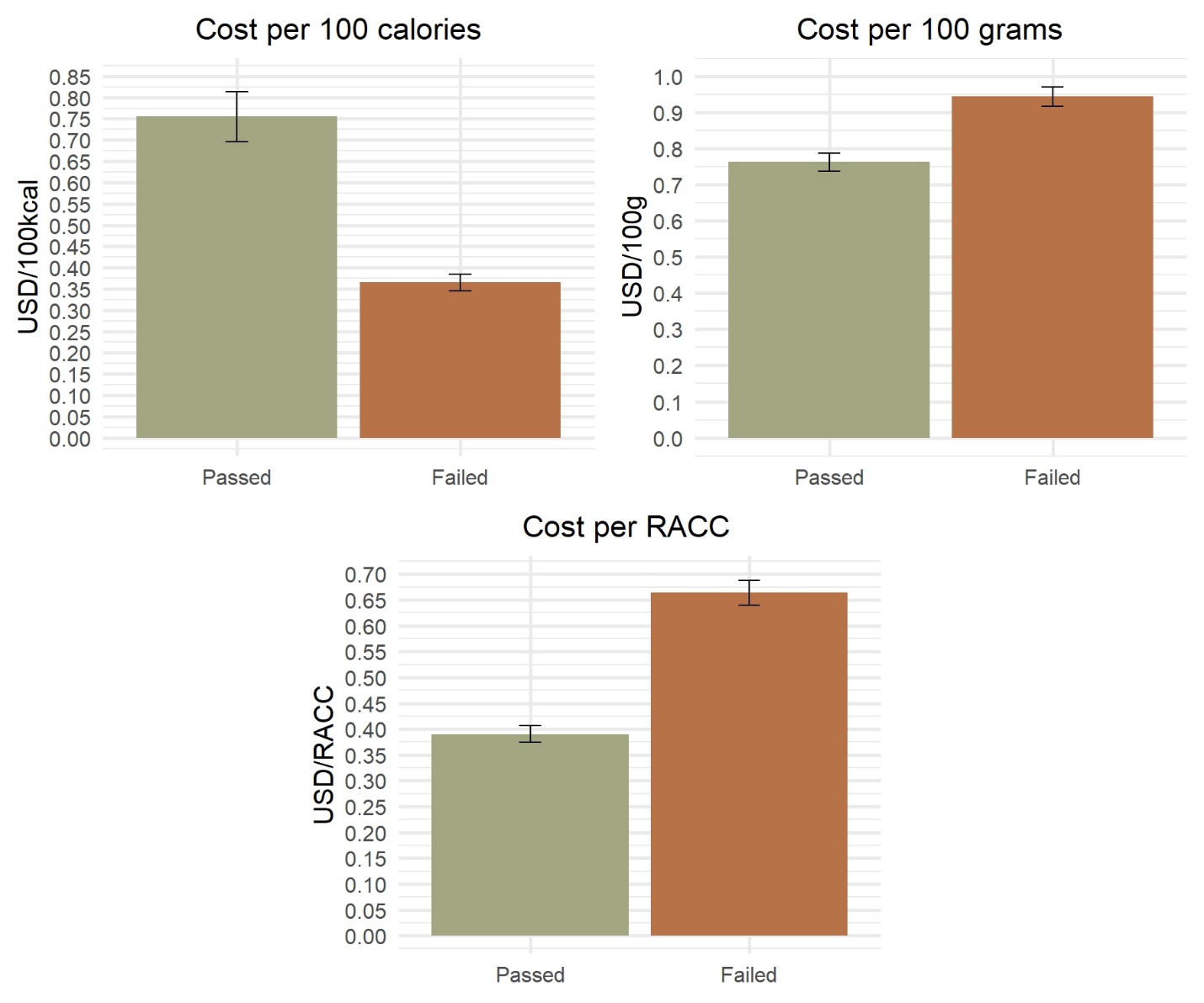


**Supplemental Figure 16**: Mean cost for the 1178 foods and beverages not included in the main analysis, resultant from the lenient Nutrients to Limit (NTL) criteria application. Data represent mean costs calculated per 100 kilocalories, 100 grams, and per serving, determined by Reference Amounts Customarily Consumed (RACC). Error bars represent the standard error of the mean for each indicator. Foods and beverages are separated by whether they passed the lenient NTL criteria. Foods and beverages that passed satisfied all three criteria for added sugar, sodium, and saturated fat, while those that failed exceeded the limits of at least one criterion.
